# Proteome-wide profiling of protein lysine acetylation in *Aspergillus flavus*

**DOI:** 10.1371/journal.pone.0178603

**Published:** 2017-06-05

**Authors:** Yangyong Lv

**Affiliations:** College of Biological Engineering, Henan University of Technology, Zhengzhou, China; University of Florida, UNITED STATES

## Abstract

Protein lysine acetylation is a prevalent post-translational modification that plays pivotal roles in various biological processes in both prokaryotes and eukaryotes. *Aspergillus flavus*, as an aflatoxin-producing fungus, has attracted tremendous attention due to its health impact on agricultural commodities. Here, we performed the first lysine-acetylome mapping in this filamentous fungus using immune-affinity-based purification integrated with high-resolution mass spectrometry. Overall, we identified 1383 lysine-acetylation sites in 652 acetylated proteins, which account for 5.18% of the total proteins in *A*. *flavus*. According to bioinformatics analysis, the acetylated proteins are involved in various cellular processes involving the ribosome, carbon metabolism, antibiotic biosynthesis, secondary metabolites, and the citrate cycle and are distributed in diverse subcellular locations. Additionally, we demonstrated for the first time the acetylation of fatty acid synthase α and β encoded by *aflA* and *aflB* involved in the aflatoxin-biosynthesis pathway (cluster 54), as well as backbone enzymes from secondary metabolite clusters 20 and 21 encoded by AFLA_062860 and AFLA_064240, suggesting important roles for acetylation associated with these processes. Our findings illustrating abundant lysine acetylation in *A*. *flavus* expand our understanding of the fungal acetylome and provided insight into the regulatory roles of acetylation in secondary metabolism.

## Introduction

Protein lysine acetylation (Kac) is a prevalent, dynamic, and reversible protein post-translational modification (PTM), important in the regulation of multiple cellular processes [1]. Kac was first discovered on histone proteins regulated by histone acetyltransferases (HATs) or histone deacetylases to affect chromatin remodeling and regulate gene expression [2,3]. However, subsequent research showed that Kac in non-histone proteins was widely distributed in almost every cellular compartment and involved in the regulation of transcription, translation, and metabolism in both prokaryotic and eukaryotic cells [4–6].

In recent years, comprehensive acetylomes were extensively studied in many prokaryotic and eukaryotic organisms, including *Escherichia coli* [5,7–9], *Bacillus subtilis* [10], *Vibrio parahemolyticus* [11], *Saccharopolyspora erythraea* [12], *Streptomyces roseosporus* [13], *Mycobacterium tuberculosis* [14], *Saccharomyces cerevisiae* [15], *Arabidopsis* [16], and rice (*Oryza sativa*) [17]. These results demonstrated that Kac occurs in numerous proteins that participate in central metabolism, protein synthesis, and secondary metabolism. For example, Kim et al. [10] identified 2803 Kac sites in 782 proteins primarily involved in central metabolism and protein synthesis. Liao et al. [13] identified 1143 Kac sites in 667 proteins from *S*. *reseosporus*, of which a non-ribosomal peptide synthase (NRPS) involved in secondary metabolism was found to be acetylated. In *S*. *cerevisiae*, acetylated proteins have been implicated in the regulation of chromatin organization, mitochondrial metabolism, and protein synthesis [15]. It is now widely accepted that Kac is an evolutionarily conserved and widespread PTM observed across different genera and families [18]. In *Aspergillus*, Kac is mainly focused on histone regulation in *Aspergillus nidulans*, where histone-3 acetylation at Lys9 or Lys14 is involved in primary metabolism [19], growth [20], and secondary metabolite production, including sterigmatocystin, terrequinone, penicillin, and orsellinic acid [21, 22]. In *Aspergillus flavus*, recent studies demonstrated that PTMs (histone methylation) are involved in morphology development and aflatoxin synthesis [23–25]; however, to the best of our knowledge, no study of the lysine acetylome in *A*. *flavus* has been reported.

*A*. *flavus*, a mostly saprophytic soil fungus and a ubiquitous and notorious pathogen, is the primary etiological agent for aflatoxin contamination of agricultural commodities, including corn, cotton, tree nuts, and peanuts [26, 27]. Genomic analysis indicated that genes encoding acetyltransferases exist in the *A*. *flavus* genome. Previous studies confirmed the importance of conserved lysine acetylation in the regulation of chromatin dynamics, gene expression and secondary metabolite production [21]. Consequently, it is important to explore the connection between lysine acetylation and aflatoxin biosynthesis in *A*. *flavus*. In comparison with the number of identified lysine-acetylated proteins found in bacteria, plants, and animals, a number of lysine-acetylated proteins in *A*. *flavus* are expected to be identified with mechanisms associated with aflatoxin biosynthesis. In this study, we presented the first systematic identification of the lysine acetylome for the aflatoxin producer *A*. *flavus* using a combination of affinity enrichment and high-resolution LC-MS/MS analysis and identified 1383 Kac sites in 652 acetylated proteins involved in various biological processes and distributed in diverse cellular locations. Additionally, 14 conserved-motif sequences surrounding Kac sites were discovered in the *A*. *flavus* acetylome. Protein-protein-interaction networks showed that Kac proteins were mainly enriched in processes associated with the ribosome, proteasome, glycolysis/gluconeogenesis, aminoacyl-tRNA biosynthesis, and oxidative phosphorylation. Specifically, our results indicated that Kac likely plays important roles in secondary metabolite biosynthesis, given our findings that enzymes involved in aflatoxin biosynthesis and two secondary metabolite clusters were found to be acetylated.

## Materials and methods

### Strain and culture conditions

*A*. *flavus* CA43, the S-strain isolates capable of producing numerous sclerotia and high concentrations of aflatoxin [28], was kindly provided by Professor Perng-Kuang Chang (Southern Regional Research Center, Agricultural Research Service, U.S. Department of Agriculture, Washington, D.C., USA). *A*. *flavus* sclerotia (1.5 × 10^6^) were inoculated onto potato dextrose agar (PDA)-cellophane plates as described previously [29] and cultivated at 30°C in the dark. The mycelia of *A*. *flavus* were harvested after 48 h cultivation for protein extraction and subsequent Kac analysis.

### Protein extraction and western blot analysis

*A*. *flavus* mycelia were frozen by liquid nitrogen and ground into a powder, followed by transfer to a 5-mL centrifuge tube and sonication three times on ice using a high-intensity ultrasonic processor (Scientz, Ningbo, China) in lysis buffer [8 M urea, 2 mM EDTA, 65 mM DTT, 30 mM nicotinamide, 3 μM trichostatin A, and 1% protease-inhibitor cocktail IV (Calbiochem; Millipore, Billerica, MA, USA)]. The remaining debris was removed by centrifugation at 20,000×*g* at 4°C for 10 min. The protein was precipitated with cold 15% trichloroacetic acid for 2 h at −20°C. After centrifugation at 20,000×*g* at 4°C for 10 min, the supernatant was discarded, and the precipitate was washed three times with cold acetone. The protein was redissolved in buffer [8 M urea and 100 mM NH_4_CO_3_ (pH 8.0)], and protein concentration was determined using a 2D Quant kit (GE Healthcare, Little Chalfont, UK) according to manufacturer instructions.

Proteins were boiled in SDS loading buffer for 3 min, subjected to 12% SDS-PAGE, and transferred to a polyvinylidene difluoride membrane. The membrane was blocked with 3% BSA at room temperature for 1 h and incubated with anti-acetyl-lysine antibody (1:1000 dilution; catalog no. PTM-101; PTM Biolabs, Hangzhou, China) in TBST buffer [25 mM Tris–HCl (pH 8.0), 125 mM NaCl, and 0.1% Tween 20] with 3% BSA. After washing three times with TBST buffer, the membrane was incubated with horseradish peroxidase-conjugated anti-rabbit antibody (1:5000 dilution) (Thermo Fisher Scientific, Waltham, MA, USA) for 1 h at 37°C. The membrane was then washed with TBST buffer and visualized with an enhanced chemiluminescence western blotting detection kit (Advansta, Menlo Park, CA, USA).

### Trypsin digestion, HPLC fractionation, and affinity enrichment of lysine-acetylated peptides

For trypsin digestion, the protein solution was reduced with 10 mM DTT for 1 h at 37°C, alkylated with 20 mM iodoacetamide for 45 min at room temperature in the dark, and then diluted four times with 100 mM NH_4_CO_3_. Trypsin was added at a 1:50 trypsin-to-protein mass ratio for the first digestion overnight and a 1:100 trypsin-to-protein mass ratio for the second 4-h digestion. The sample was fractionated by high-pH, reverse-phase HPLC using an Agilent 300Extend C18 column (5-μm particles, 4.6-mm internal diameter, 250-mm length; Agilent, Santa Clara, CA, USA). Briefly, peptides were first separated using a gradient of 2% to 60% acetonitrile in 10 mM NH_4_CO_3_ (pH 10) over 80 min into 80 fractions. The peptides were combined into three fractions and dried by vacuum centrifugation.

To enrich lysine-acetylated peptides, 4 mg trypsinized peptides dissolved in NETN buffer [100 mM NaCl, 1 mM EDTA, 50 mM Tris–HCl, and 0.5% NP-40 (pH 8.0)] were used for each immunoprecipitation experiment. The dissolved peptides were incubated separately with 20μl agarose beads coupled to anti-acetyl-lysine antibody PTM-104 (Jingjie PTM Bio, Hangzhou, China) and ICP0388 (Immunechem Pharmaceuticals, Burnaby, Canada) at 4°C overnight with gentle shaking. Then the flow-through of the two immunoprecipitation experiments was then combined and subjected to immunoprecipitation with agarose beads coupled to PTM-104 (20μl) and ICP0388 (20μl) together to obtain more fully enriched acetylated peptides.

The beads were washed four times with NETN buffer and twice with ddH_2_O. The bound peptides were eluted from the beads with 0.1% trifluoroacetic acid, vacuum-dried, and the obtained peptides were cleaned with C18 ZipTips (Millipore) according to manufacturer instructions, followed by LC-MS/MS analysis.

### LC-MS/MS analysis

Peptide separation was performed using a reversed-phase analytical column (Acclaim PepMap 100; Thermo Fisher Scientific, Raleigh, NC, USA). First, peptides were dissolved in 0.1% formic acid and loaded onto a reversed-phase pre-column. A constant flow rate of 280 nL/min was established with an EASY-nLC 1000 ultra-performance liquid chromatography (UPLC) system (Thermo Fisher Scientific), with a gradient consisting of increases from 6% to 23% solvent B (0.1% FA in 98% acetonitrile) for 24 min, 22% to 35% solvent B for 8 min, and climbing to 80% solvent B for 4 min before holding at 80% for the final 4 min.

Peptides were subjected to nanoelectrospray ionization, followed by MS/MS analysis using a Q Exactive Plus system (Thermo Fisher Scientific) coupled online with the UPLC system. Intact peptides were detected in the orbitrap at a resolution of 70,000 and selected for MS/MS using a normalized collision-energy setting of 30. Ion fragments were detected in the orbitrap at a resolution of 17,500. A data-dependent procedure that alternated between one MS scan, followed by 20 MS/MS scans was applied for the top 20 precursor ions above a threshold ion count of 1E4 in the MS-survey scan, with a 10-s dynamic exclusion. The electrospray voltage applied was 2.0 kV, and automatic gain control was used to prevent overfilling of the ion trap, resulting in 5E4 ions accumulated for generation of MS/MS spectra. For MS scans, the m/z scan range was 350 to 1800. The mass spectrometry proteomics data from three immunoprecipitation experiments were deposited to the ProteomeXchange Consortium via the PRIDE [30] partner repository with the dataset identifier PXD004802.

### Database search

The resulting MS/MS data was processed using MaxQuant [31] with an integrated Andromeda search engine (v.1.4.1.2; http://www.biochem.mpg.de/5111795/maxquant). MS/MS spectra were searched against the Uniprot *A*. *flavus* database (v.2015.9.21; http://www.uniprot.org/) concatenated with a reverse decoy database. Trypsin/P was specified as the cleavage enzyme, and the search allowed for up to three missing cleavages, four modifications per peptide, and five charges. Mass error was set to five ppm for precursor ions and 0.02 Da for fragment ions. Carbamidomethylation on Cys was specified as a fixed modification, and oxidation on Met, acetylation on Lys, and acetylation on protein N-termini were specified as variable modifications. All other parameters in MaxQuant were set to default values. False-discovery rate (FDR) thresholds for proteins, peptides, and modification sites were specified at 1%. Minimum peptide length was set at 7, and the site-localization probability was set at > 0.75. The score cut-off that was used for modification of acetylated peptides was at ≥ 40.

### Bioinformatics analysis

#### Functional annotation and enrichment analysis of acetylated proteins

Gene Ontology (GO; http://www.geneontology.org) annotation and Kyoto Encyclopedia of Genes and Genomes (KEGG; http://www.genome.jp/kegg/pathway.html) analyses were performed according to previously reported methods [32]. A domain annotation was performed using InterProScan on the InterPro domain database (http://www.ebi.ac.uk/interpro/) via web-based interfaces and services [33]. The subcellular localization was determined by Wolfpsort (version of PSORT/PSORT II; http://psort.hgc.jp/).

GO, KEGG pathway, and protein domain-enrichment analyses were performed, and for each category, a two-tailed Fisher’s exact test was employed to test the enrichment of the identified protein against all database proteins. Correction for multiple-hypothesis testing was performed using standard FDR-control methods. The GO, KEGG pathway, and protein-domain results with a corrected *p* < 0.05 were considered significant.

#### Motif discovery and clustering analysis

Soft motif-X [34] was used to analyze the models of sequences with amino acids in specific positions of modify-21-mers (10 amino acids upstream and downstream of the site) for all protein sequences. All protein sequences from databases were used as background-database parameters, while other parameters were set to the defaults. All motifs with *p* < 0.05 were enriched in each subcellular compartment, and a matrix composed of enriched results was generated. The filtered matrix was transformed by the function x = −log10 (*p* value), and the values were z-transformed for each category. Cluster membership was visualized by a heat map using the “heatmap.2” function from the “gplots” R-package.

#### Hierarchical clustering analysis of motifs and pathways

For hierarchical clustering based on acetylation motifs and protein-pathway enrichment, motifs and proteins from the categories were obtained following enrichment respectively and then the categories were filtered to identify those that were enriched in at least one of the clusters with p-value < 0.05. Cluster membership was visualized using a heat map via the “heatmap.2” function from “gplots” R-package.

#### Protein-protein-interaction network analysis

The acetylated protein-protein-interaction network was obtained from the STRING database (http://string.embl.de/), which defined interaction confidence as ≥ 0.7 (high confidence). The interaction networks of acetylated proteins were visualized using Cytoscape software (http://www.cytoscape.org/) [35].

## Results and discussion

### Identification and analysis of lysine-acetylated proteins in *A*. *flavus*

**Fig 1:**
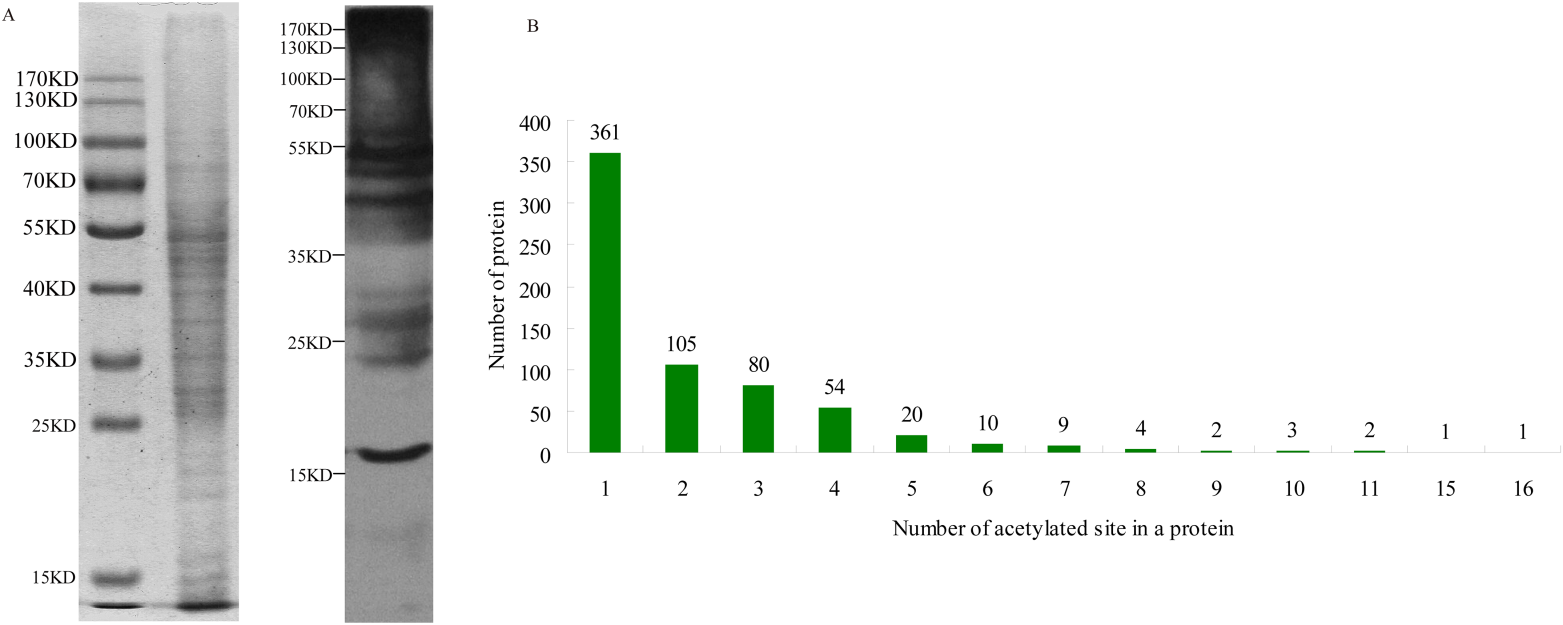
Lysine acetylation status is analyzed by using SDS-PAGE and western blotting. (**A**) Confirmation of acetylated proteins present in *A*. *flavus*. 15μg protein samples were loaded for SDS-PAGE analysis. Acetylated Lys antibody (PTM Biolabs) was used in a 1:1000 dilution. (**B**) Distribution of lysine-acetylated peptides based on the number of acetylation sites.

To evaluate the extent of acetylation in the *A*. *flavus* proteome, protein extracts derived from *A*. *flavus* CA43 grown on PDA medium were subjected to SDS-PAGE and western blot analysis using an anti-acetyl-lysine antibody (Fig 1). As shown in Fig 1, proteins with different molecular weights visualized by Coomassie staining demonstrated strong reactivity with the anti-acetyl-lysine antibody, indicating that abundant Kac was present in diverse *A*. *flavus* proteins.

To gain further insight into the large-scale dataset of acetylated proteins in this aflatoxin producer, immuno-affinity enrichment and MS-based proteomics approaches using nano-LC were employed to identify Kac in *A*. *flavus* CA43 (S1 Fig). The distribution of mass errors associated with the most frequently identified peptides was < 0.02 Da, indicating that the mass accuracy of the MS data fit the requirement, and that the length of most peptides was between 8 and 20 residues, agreeing with properties of trypsinized peptides (S1 Fig) and implying that the sample preparation met the quality threshold. Of the obtained 5233 reliable peptides (score≧40), 1413 peptides were acetylated (Acetyl [K] site IDs in peptides are listed in S1 Table). Of the 3820 non-acetylated peptides, missed cleavages occurred in 449 (missed cleavages in peptides are listed in S1 Table) accounting for 11.75% of the non-acetylated peptides. A total of 1383 Kac sites in 652 proteins were identified based on the data of the three immunoprecipitation experiments (S1 Table), indicating that at least 5.18% of the proteins in *A*. *flavus* were acetylated under the analyzed conditions.

To investigate the distribution of the identified modification sites in the *A*. *flavus* proteins, the number of Kac sites per protein was calculated (Fig 1). Our findings showed that 55.37% of the proteins contained only one acetylation site, while 44.63% of the proteins were acetylated at multiple lysines (Fig 1 **and** S1 Table). Of the Kac proteins, 52 contained five or more Kac sites, and seven had at least 10 Kac sites. Mitochondrial aconitate hydratase (B8N211, also named aconitase and involved in the citrate cycle) was the most intensively acetylated protein, with 15 acetylation sites. Three examples of MS/MS spectra of acetylated peptides are shown in S2 Fig. All the MS/MS data have been deposited on ProtomeXchange Consortium. These data provided the first global survey of lysine acetylation in *A*. *flavus*.

### Analysis of Kac sites in *A*. *flavus*

**Fig 2:**
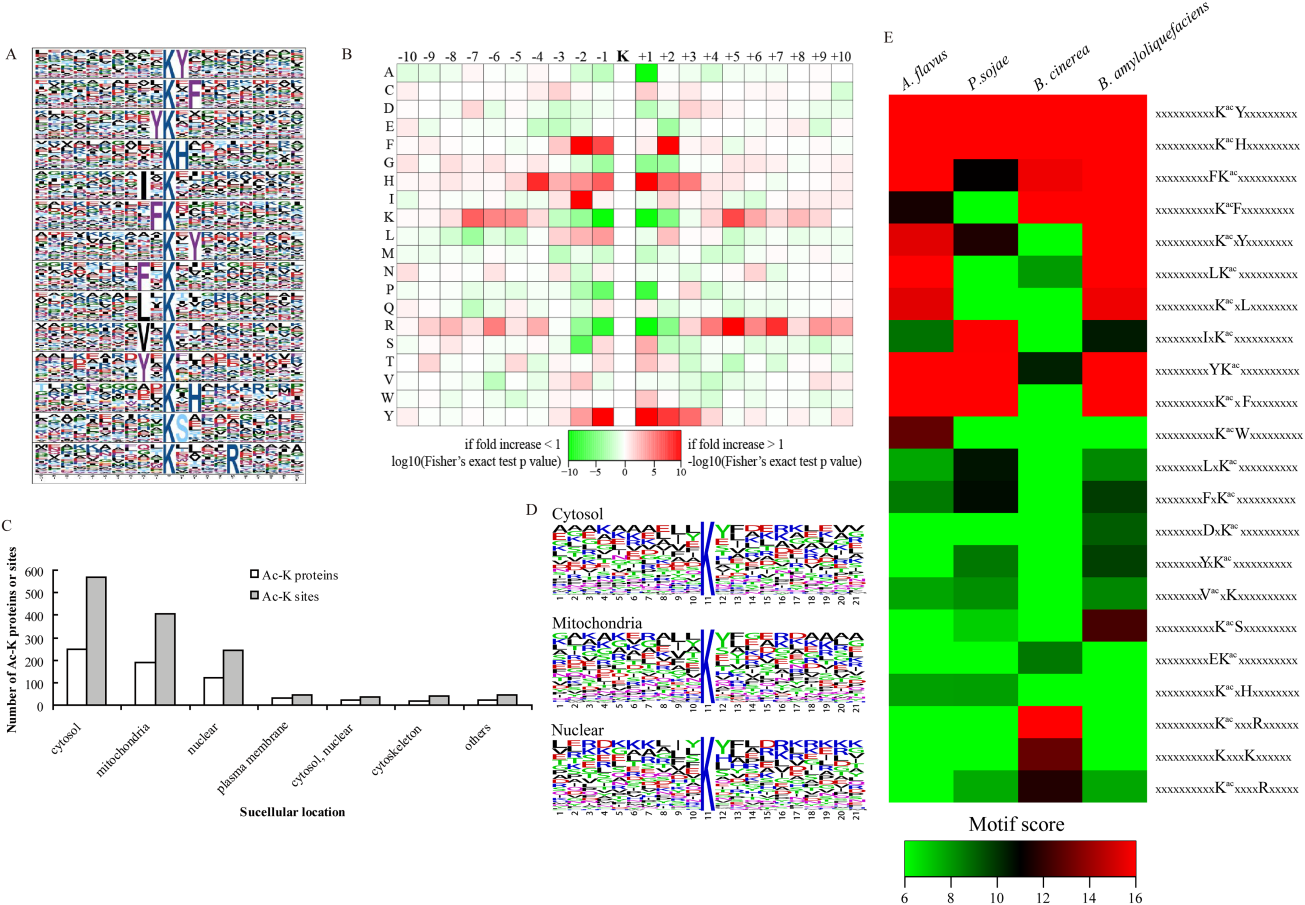
Motif analysis of Kac peptides. (**A**) Acetylation motifs and conservation of acetylation sites. The height of each letter corresponds to the frequency with which that amino acid residue is found at that position. (**B**) Heat map representing the amino acid composition of the Kac sites, showing the frequency of the different types of amino acids surrounding Kac sites. (**C**) Cellular distribution of acetylated proteins and sites. (**D**) Sequence logo plots of normalized amino acid frequencies ±10 amino acids from the lysine acetylation site in cellular compartments. (**E**) Comparison analysis of acetylation motifs between *Aspergillus flavus*, *Phytophthora sojae*, *Botrytis cinerea*, and *Bacillus amyloliquefaciens*.

To elucidate the properties of amino acids surrounding identified acetylation sites in *A*. *flavus* proteins, motif-X was employed to search for occurrences of amino acid motifs (10 amino acids upstream and downstream of the acetylation site) in identified lysine-acetylated proteins (Fig 2). Of all acetylated-lysine peptides, 1128 were matched to 13 conserved motifs, including xxxxxxxxxx K^ac^Yxxxxxxxxx, xxxxxxxxxx K^ac^xFxxxxxxxx, xxxxxxxxx YK^ac^xxxxxxxxxxx, xxxxxxxxxxK^ac^Hxxxxxxxxx, xxxxxxxxIxK^ac^xxxxxxxxxx, xxxxxxxxxFK^ac^xxxxxxxxxx, xxxxxxxxxxK^ac^xYxxxxxxxx, xxxxxxxxFxK^ac^xxxxxxxxxx, xxxxxxxxLxK^ac^xxxxxxxxxx, xxxxxxxxVxK^ac^xxxxxxxxxx, xxxxxxxxYxK^ac^xxxxxxxxxx, xxxxxxxxxxK^ac^xHxxxxxxxx, xxxxxxxxxxK^ac^Sxxxxxxxxx, and xxxxxxxxxxK^ac^xxxxRxxxxx (where K^ac^ indicates the acetylated lysine, and ‘x’ indicates a random amino acid residue), which constituted more conserved motifs than those previously reported from other microorganisms (Fig 2A **and** S2 Table). A survey of these motifs suggested that two types of residues were observed in areas surrounding the acetylated lysines. The first was tyrosine (Y), isoleucine (I), phenylalanine (F), leucine (L), and valine (V) upstream of Kac sites, and the second was tyrosine (Y), phenylalanine (F), histidine (H), and arginine (R) downstream of Kac sites. According to the position of the residues in proximity to the acetylated lysine, most of the conserved residues were located at the ±1 or ±2 positions of the Kac sites (F, I, L, V, and Y), similar to patterns observed in *O*. *sativa* [6], except for the R at the +5 position (Fig 2B). Interestingly, two acetylated-lysine motifs (K^ac^H and K^ac^Y) were observed in *E*. *coli* [36], the human pathogen *M*. *tuberculosis* [14], the secondary metabolite producer *S*. *erythraea* [12], and *S*. *roseosporus* [13], *O*. *sativa* [6], the marine bacterium *V*. *parahaemolyticus* [11], and human cells [37]. Our results also showed that F and Y were the most conserved amino acids detected in both upstream and downstream regions from the acetylated lysines (Fig 2A). The essential and conserved amino acid F, a precursor of several secondary metabolites, was also enriched in *O*. *sativa* [6] and *V*. *parahaemolyticus* [11]. These results were consistent with previous findings regarding conserved motifs and amino acids and could indicate the importance of lysine acetylation in microorganisms, plants, and animals.

To better understand the characteristics of protein acetylation motifs in *A*. *flavus*, comparison of hierarchical clustering results with the recent published results from other organisms, including filamentous fungi *Phytophthora sojae* [38] and *Botrytis cinerea* [39] and the antibiotic producer *Bacillus amyloliquefaciens* [40], was conducted (Fig 2E). The motif comparison analysis indicated that two conserved motifs xxxxxxxxxxK^ac^Yxxxxxxxxx and xxxxxxxxxxK^ac^Hxxxxxxxxx, discussed previously were also enriched in the other microorganisms, suggesting the conservation of these motifs. In comparison with the other two filamentous fungi, we observed three specific motifs xxxxxxxxxxK^ac^ xYxxxxxxxx, xxxxxxxxxLK^ac^ xxxxxxxxxx, and xxxxxxxxxxK^ac^xLxxxxxxxx in *A*. *flavus*. Interestingly, these three motifs were also enriched in the secondary metabolite producer *B*. *amyloliquefaciens*, which might suggest their specificity in secondary metabolite-producing strains, although their detailed roles still need to be investigated.

The distribution of different motifs and the number of Kac sites in cellular compartments was also assessed to profile the characterization of amino acids surrounding identified acetylation sites. Kac proteins and sites are thought to be predominantly distributed in nuclear and mitochondrial [41] and we also found a large number of Kac proteins in these compartments. However, Kac proteins in cytosol were also highly represented in the acetylome (Fig 2C), which was similar to that in *S*. *cerevisiae* [41]. The analysis of local sequence context around the acetylation sites showed that L and Y were enriched in the -1 and +1 positions. Cytosol and mitochondria acetylation motifs are similar, but different from nuclear motifs (Fig 2D). In cytosol and mitochondria proteins, there was a preference for L in both −1 and −2 positions. Glycine at −1 found in human cells [42] and *S*. *cerevisiae* [41] was not observed in specific proteins here.

### Functional annotation and subcellular localization of lysine-acetylated proteins in *A*. *flavus*

**Fig 3:**
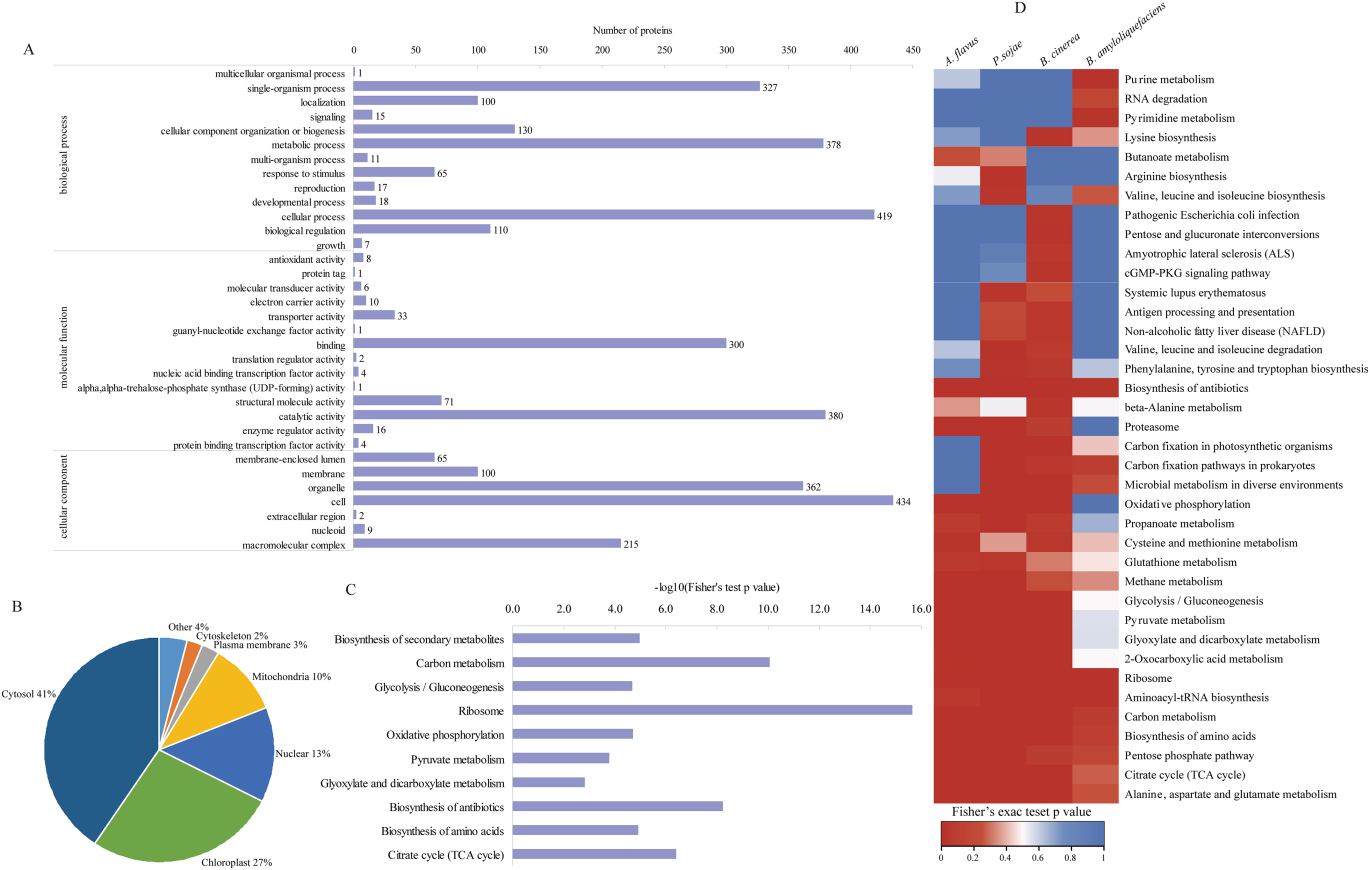
**(A)** GO, **(B)** subcellular-localization analysis, and **(C)** KEGG-pathway enrichment of the identified Kac proteins. (**D**) Comparison analysis of enriched pathways between *Aspergillus flavus*, *Phytophthora sojae*, *Botrytis cinerea*, and *Bacillus amyloliquefaciens*.

To better understand the lysine acetylome in *A*. *flavus*, GO and subcellular-localization analysis were performed (S3 Table). With respect to biological processes, 419 proteins were involved in cellular processes, 378 in metabolic processes, 327 in single-organism processes, 130 in cellular-component organization or biogenesis, 110 in biological regulation, and 100 in subcellular localization. According to molecular function, 380 acetylated proteins were involved in catalytic activity and 300 in binding activities. As for cellular components, 434 proteins were cell proteins, 363 were organelle proteins, 215 were macromolecular-complex proteins, and 100 were located in the membrane (Fig 3A). Within the classification of subcellular localization, 41% of the identified lysine-acetylated proteins localized to the cytosol, 27% to chloroplasts, 13% to the nucleus, 10% to the mitochondria, 3% to the plasma membrane, and 2% to the cytoskeleton (Fig 3B).

Additionally, we conducted KEGG-pathway, GO, and protein domain-enrichment analyses (S3 Table). KEGG metabolic pathway enrichment demonstrated that acetylation occurred on many proteins involved in functions associated with the ribosome, carbon metabolism, and the biosynthesis of antibiotics, secondary metabolites, and amino acids, as well as the citrate cycle and glycolysis/gluconeogenesis (Fig 3C). GO-enrichment analysis indicated that the acetylated proteins were markedly enriched in structural molecular activity, functions associated with the ribosome, and intracellular and metabolic processes (S3 Fig). Protein domain-enrichment analyses showed that protein domains, including the N-termini of nucleophile aminohydrolases, translation protein SH3-like domains, and ribosomal protein L2-domain 2, were mainly enriched. Additionally, ATPase, (F1/V1/A1 complex, α/β subunit) was also enriched, consistent with results observed for *O*. *sativa* [6] (S3 Fig). These findings demonstrated that Kac play important roles in the most fundamental cellular processes.

To better illustrate the characteristics of pathway enrichment in *A*. *flavus*, we conducted a comparison of hierarchical clustering analysis with recently published findings (Fig 3D). KEGG pathway analysis showed that the acetylated proteins enriched in species-specific pathway of *A*.*flavus* were not observed. In the three kinds of filamentous fungi, Kac proteins mainly participated in energy supply, carbon metabolism, glycolysis, the citrate cycle, the pentose phosphate pathway, and metabolism of amino acids were enriched, which was consistent with previous findings [43]. However, in comparison with filamentous fungi, the Kac proteins enriched in glycolysis, gluconeogenesis and 2-oxocarboxylic acid metabolism in *B*. *amyloliquefaciens* were not significantly presented, with the reasons for this requiring further elucidation.

### Protein-interaction network analysis

To understand the cellular processes regulated by acetylation in *A*. *flavus*, we generated a protein-interaction network for all acetylated proteins using Cytoscape. The results showed that 457 acetylated proteins were classified as network nodes connected by 3970 direct interactions (S4 Fig **and** S4 Table). Four sub-networks, including those describing glycolysis/gluconeogenesis, aminoacyl-tRNA biosynthesis, oxidative phosphorylation, proteasome activity, and ribosome activity, were especially enriched (S5 Fig). Our observation of sub-networks enriched for ribosome activity, glycolysis/gluconeogenesis, and aminoacyl-tRNA biosynthesis was consistent with previous reports, suggesting the conservation of Kac-related involvement in a wide range of regulatory components in prokaryotes and eukaryotes [6, 11, 12, 41].

### Involvement of Kac in different cellular processes

Protein Kac has emerged as a key PTM capable of regulating gene expression in different cellular metabolic parthways through modification of core histones to remodel the chromatin and result in alternation of protein–protein, protein–DNA and protein–RNA interactions, as well as through modification of proteins to alter their stability and activity [42]. In *Aspergillus*, chromatin structure and nucleosome modifications play vital roles in gene expression, with flexibility of the chromatin structure regulated by modifying specific histone residues with different PTMs [44,45]. In this study, we found that histones H1, H2A, H2B, H3, and H4 were acetylated, including six Lys residues in both histone H3 (B8N4Q2) and H4 (B8N4Q3) (S1 Table). Covalent PTMs commonly occurred at histones H3 and H4 [44], which were inconsistent with our data. Acetylation of core histones could loosen chromatin structure and correlate with gene activation [42]. Additionally, similar to *S*. *cerevisiae* [41], the chromatin-remodeling complex (B8NPF4) was also found to be acetylated, which also might also be involved in chromatin alternation.

Transcription factors, together with RNA polymerase II, promote basal gene transcription. Previous studies showed that transcription factor acetylation was involved in the regulation of gene expression and metabolic homeostasis [46]. In mammalian cells, acetylation of RNA polymerase II regulates growth-factor-induced gene transcription [47]. In *A*. *flavus*, the functions associated with transcriptional factor acetylation have not been characterized. Our results showed that 21 transcriptional factors, including 6 families, were acetylated (S1 Table) and their function involving metabolic processes require further study. Additionally, we also observed that RNA polymerase II subunit 7 (B8MXT0) was acetylated, which is in agreement with reports associated with *S*. *roseosporus* [13]. Furthermore, proteins involved in translational regulation, including translation-elongation factors, ribosomal proteins, and tRNA synthetases, were lysine-acetylated, which is inconsistent with previous findings in *E*. *coli* [36].

Additionally, HATs (B8NNR7 and B8N0P8) responsible for histone Kac were also acetylated, which was consistent with results from *S*. *cerevisiae* [41]. In mammalian cells, transcriptional coactivator p300 is a HAT, and its autoacetylation is a well-established example of Lysine acetyltransferases (KAT) acetylation that regulates its own enzymatic activity [48]. Our data demonstrated that extensive acetylation of KATs is a conserved and general property ranging from microbiology to mammalian cells. Previous studies demonstrated that acetylation consistently occurred by enzyme-catalyzed reactions [49] or non-enzyme catalyzed reactions [8]. Generally, the acetyltransferases participated in the enzyme-catalyzed reactions. However, it remains unclear how many protein acetyltransferases are involved in these reactions in *A*. *flavus*. An individual acetyltransferase can modify several substrates, and one protein can be acetylated by multiple acetyltransferases [12]. Hence, determination of which protein acetyltransferases modify these various proteins remains to be investigated in future studies. In addition to the enzyme-catalyzed acetylation, nonenzymatic acetylation can also result in lysine modification [50], which plays different roles according to different species [51,52]. In *E*. *coli*, recent study showed that levels of acetyl-phosphate (AcP), a high-energy intermediate of the phosphotransacetylase-acetate kinase (Pta-AckA) pathway [53], are correlated with acetylation levels, suggesting that AcP may acetylate proteins non-enzymatically [8]. Further study showed that acP-dependent acetylation is both non-enzymatic and specific, with specificity determined by the accessibility, reactivity and three-dimensional microenvironment of the target lysine [9]. However, the Pta-AckA pathway and AcP metabolism in *Aspergillus* remain to be investigated. 14-3-3 proteins are widely expressed proteins that specifically bind to phosphoserine or phosphothreonine, thereby regulating a diverse set of cellular processes, such as signal transduction, cell cycle progression, and DNA damage repair [54]. In *S*. *cerevisiae*, sitemutation analysis indicted that acetylation of 14-3-3 proteins impaired its phosphorylation-dependent interactions [41]. In *A*. *flavus*, two kinds of 14-3-3 family proteins, including B8N2H5 (Kac at 119,124) and B8NLM9 (Kac at 82, 117, and 105), were found to be acetylated, which indicated that similar function might exist. In human cells, acetylation of DNA damage repair protein p53 regulates its stability, modulates interactions with TATA-box binder protein–associated factor, and regulates its transcriptional activity [55]. In *S*. *cerevisiae*, Ku70 and Ku80 involved in DNA damage repair are acetylated [41]. In our work, DNA damage response protein (B8N1R8) was found to be acetylated, and its activity might be affected.

S-adenosyl-methionine synthase (MAT; also called methionine adenosyltransferase) catalyzes the formation of S-adenosyl-L-methionine (SAM) and is well-conserved from bacteria to eukaryotes [56]. Previous studies showed that SAM is highly reactive and participates in numerous metabolic pathways, including the methionine cycle, and polymine formation [57]. Moreover, as a methyl donor in intracellular reactions, SAM transfers methyl group to various acceptors, including nucleic acids, proteins, and lipids, as well as to precursor molecules, such as catecholamines, guanidinoacetate, and other biogenic amines [58]. In mammals, the role of MAT in the therapeutic application of diseases has been concentrated [59]. In *E*. *coli*, MATdeficient strains exhibited growth deficiencies and celldivision defects, and acetylation of MAT leads to a decrease in enzymatic activity [60]. In *A*. *flavus*, enzymatic activity of SAM synthase (B8NJU1) might be changed owing to the presence of four Kac sites; however, its function in *A*. *flavus* remains to be elucidated.

In *A*. *flavus*, growth-related proteins, including hyphal growth and nutrition-absorption factors, were also lysine acetylated. Tubulin heterodimers (α/β-tubulin) are the building block of microtubules, which are major elements of the cytoskeleton. A previous study showed that acetylation lysine 40 of α-tubulinis associated with stable microtubule structures such as axonemes [61]; however, functions of β-tubulin remain to be investigated. In this study, acetylation of β-tubulin (B8NJQ2) at K75 might also affected microtubule structure. Additionally, NmrA, a transcriptional regulator involved in PTM of the GATA-type transcription factor AreA controlling nitrogen metabolite repression [62], was also lysine acetylated. The Rho GTPase Rho1 [63], G-protein γ-subunit [64], and ubiquitin [65] in *A*. *nidulans* and all involved in regulating cell growth were observed as having been lysine acetylated in *A*. *flavus*.

**Fig 4:**
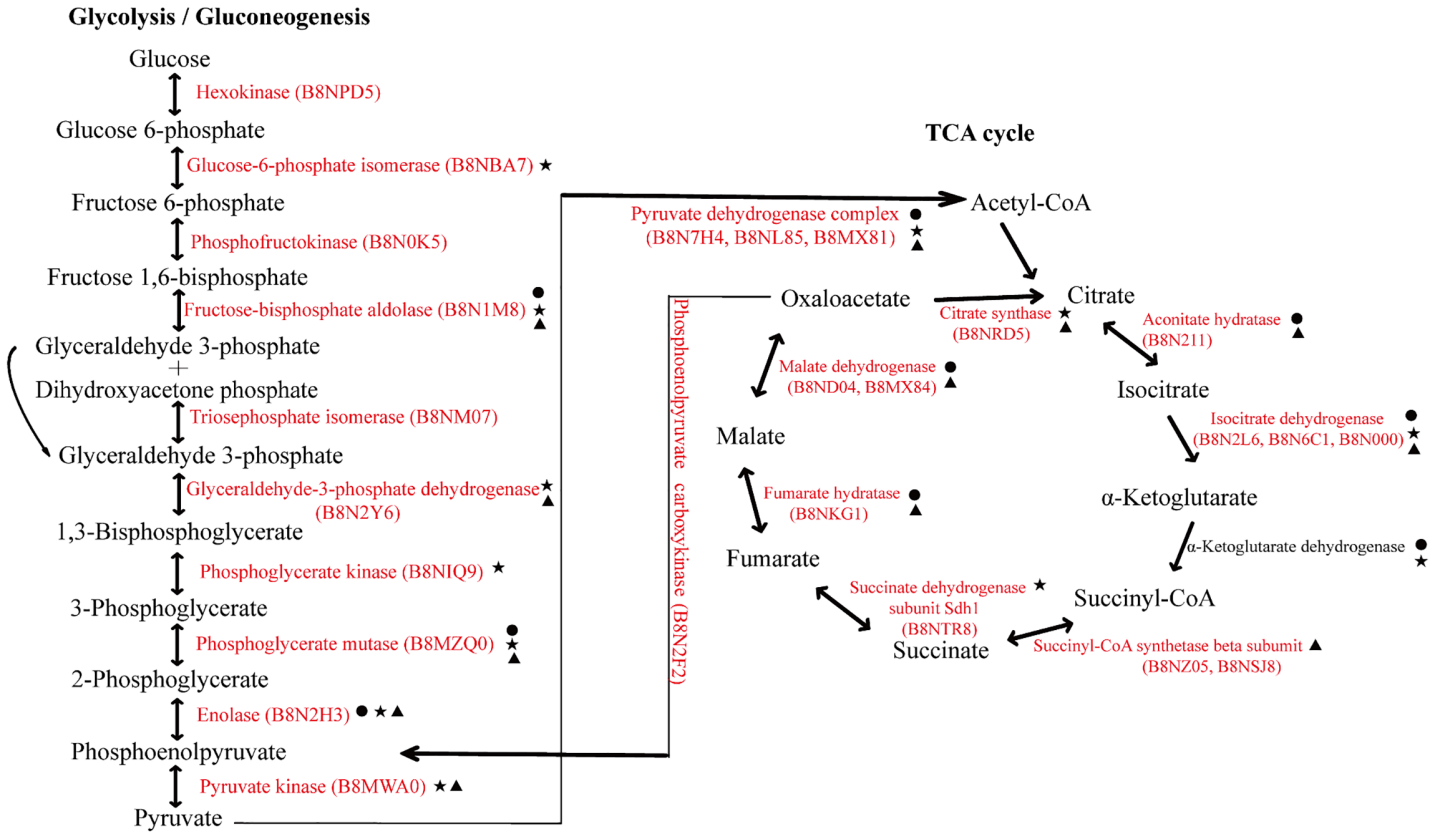
Acetylation of metabolic enzymes identified as involved in glycolysis/gluconeogenesis and the citric acid cycle. The identified numbers of lysine-acetylated enzymes and proteins are shown in red. The identified Kac proteins found in mammalian cells are marked with●, those in *Escherichia coli* with ★, and those in *Saccharopolyspora erythraea* with▲.

Several enzymes involved in central metabolic pathways were acetylated in *A*. *flavus* (Fig 4). In the glycolysis pathway, ten glycolytic enzymes required for catalyzing key reactions in the conversion of glucose to pyruvate were subjected to Kac, while seven enzymes involved in the citric acid cycle were lysine acetylated in *A*. *flavus* (Fig 4). In mammalian samples, three enzymes (fructose-bisphosphate aldolase, phosphoglycerate mutase, and enolase) involved in glycolysis and five enzymes (aconitate hydratase, isocitrate dehydrogenase, α-ketoglutarate dehydrogenase, fumarate hydratase, and malate dehydrogenase) involved in the citric acid cycle were also found to be lysine acetylated [37]. In *E*. *coli*, seven enzymes (glucose-6-phosphate isomerase, fructose-bisphosphate aldolase, glyceraldehyde-3-phosphate dehydrogenase, phosphoglycerate kinase, phosphoglycerate mutase, enolase, and pyruvate kinase) involved in glycolysis and four proteins (citrate synthase, isocitrate dehydrogenase, α-ketoglutarate dehydrogenase, and succinate dehydrogenase) involved in the citric acid cycle were lysine acetylated [36]. In *S*. *erythraea*, five enzymes (fructose-bisphosphate aldolase, glyceraldehyde-3-phosphate dehydrogenase, phosphoglycerate mutase, enolase, and pyruvate kinase) involved in glycolysis and six enzymes (citrate synthase, aconitate hydratase, isocitrate dehydrogenase, succinyl-CoA synthetase, fumarate hydratase, and malate dehydrogenase) involved in the citric acid cycle [12] were detected as having been lysine acetylated. Additionally, pyruvate dehydrogenase, the enzyme responsible for converting pyruvate to acetyl-CoA and producing an NADH molecule, was also lysine acetylated in *A*. *flavu*s, mammalian cells [37], *E*. *coli* [36], and *S*. *erythraea* [12]. The consistency observed in the acetylation of these enzymes across species suggests potentially conserved functions related to modified regulation of metabolic flux in prokaryotes and eukaryotes. However, acetylated hexokinase, phosphofructokinase, triosephosphate isomerase, and phosphoenolpyruvate carboxykinase in *A*. *flavus* were not reported in mammalian cells, *E*. *coli*, or *S*. *erythraea*.

Previous studies demonstrated that lysine acetylation is a prevalent modification in enzymes that catalyze intermediate metabolism. In human liver cells, almost every enzyme in glycolysis, gluconeogenesis, the tricarboxylic acid cycle, the urea cycle, fatty acid metabolism, and glycogen metabolism was found to be acetylated and the concentration of metabolic fuels, such as glucose, amino acids, and fatty acids, influenced the acetylation status of metabolic enzymes [43]. Additionally, the Kac profile of the metabolic enzymes in *S*. *enterica* was modified in response to different carbon sources [66]. Furthermore, in *S*. *enterica*, activity of acetyl-CoA synthetase (ACS) was controlled through reversible acetylation as an on-off switch [67]. In *A*. *flavus*, formation of the aflatoxin occurs in two phases beginning with the formation of a hexanoyl starter unit catalyzed by fatty acid synthases using acetyl-CoA and malonyl-CoA as precursors, followed by subsequent extension by a polyketide synthase [68]. Previous studies showed that the activity of metabolic enzymes and thus the metabolic flux were modified though lysine acetylation [43, 66, 67]. These results indicated that lysine acetylation of pyruvate dehydrogenase might affect the activities of the enzymes involved in the precursor-supplied pathways and regulate the metabolic flux for the biosynthesis during aflatoxin biosynthesis. Additionally, the other acetylated enzymes involved in glycolysis and citric acid cycle might also regulate metabolic flux, confirming that Kac is important in maintaining the energy balance in the cell [69].

### Clusters of proteins involved in secondary metabolism

*A*. *flavus* is a saprophytic aerobic fungus notorious for carcinogenic mycotoxin and aflatoxin production. Here, we assessed the effects of lysine acetylation in the regulation of secondary metabolite production, particularly aflatoxin biosynthesis. Our results indicated that the first two enzymes encoded by *aflA* and *aflB*, fatty acid synthase α (B8NL81) and fatty acid synthase β (B8NL80) directly involved in aflatoxin biosynthesis, respectively, were acetylated (Table 1 **and** S6 Fig) at four Kac sites at Lys152, Lys882 (S2 Fig), Lys1285 (S6 Fig), and Lys1970 in B8NL80 and nine Kac sites at Lys64, Lys168, Lys343, Lys504 (S6 Fig), Lys758, Lys930, Lys1041, Lys1066, and Lys1590 in B8NL81. In *A*. *flavus*, norsolorinic acid is the first stable precursor of aflatoxin and can be converted into sterigmatocystin and aflatoxin by subsequent enzyme catalysis [70]. In the aflatoxin pathway, biosynthesis of norsolorinic acid relies upon expression of *aflA* (AFLA_089170) and *aflB* (AFLA_089160) [71]. Previous studies proposed that enzymes, including nonribosomal peptide synthetases involved in antibiotic biosynthesis, could be regulated by acetylation modification [72]. Such observations indicated that reversible acetylation might have a direct regulatory role in aflatoxin biosynthesis in *A*. *flavus*. Although the effect of Kac on the first two enzymes involved in the aflatoxin pathway remain to be investigated, our findings implied that they might hold potential importance in the regulation of aflatoxin biosynthesis.

**Table 1:** Protein information involved in synthesis of aflatoxin in *A*. *flavus*.

Proteins	Protein names	UniProt Accession	Gene Accession
aflA	Fatty acid synthase alpha subunit FasA	B8NL81	AFLA_089170
aflB	Fatty acid synthase beta subunit	B8NL80	AFLA_089160
aflC	polyketide synthase	B8NI04	AFLA_139410
aflD	reductase	B8NI02	AFLA_139390
aflE	Norsolorinic acid reductase	B8NHZ5	AFLA_139310
aflF	dehydrogenase	B8NI07	AFLA_139440
aflG	cytochrome P450 monooxygenase	B8NHZ0	AFLA_139260
aflH	short chain alcohol dehydrogenase	B8NHZ7	AFLA_139330
aflI	Averufin oxidase A	B8NHY7	AFLA_139230
aflJ	esterase	B8NHZ6	AFLA_139320
aflK	VERB synthase	B8NHY3	AFLA_139190
aflL	desaturase/ P450 monooxygenase	B8NHY9	AFLA_139250
aflM	dehydrogenase/ ketoreductase	B8NHZ4	AFLA_139300
aflN	monooxygenase	B8NHZ2	AFLA_139280
aflY	monooxygenase/ oxidase	B8NHY0	AFLA_139160
aflO	Demethylsterigmatocystin 6-O-methyltransferase	B8NHY6	AFLA_139220
aflP	Sterigmatocystin 8-O-methyltransferase	B8NHY5	AFLA_139210
aflQ	Cytochrome P450 oxidoreductase OrdA-like	B8N6X7	AFLA_017300

In addition to lysine-acetylated proteins involved in the aflatoxin pathway, several proteins involved in other secondary metabolite clusters were also found to be acetylated. In *A*. *flavus*, 55 secondary metabolite clusters were predicted by the web-based software Secondary Metabolite Unknown Region Finder (SMURF; http://www.jcvi.org/smurf) [73], and to date, only three clusters, including those associated with aflatoxin (cluster 54), cyclopiazonic acid (cluster 55), and aflatrem (cluster 32) production, have been characterized [74,75]. In this study, two enzymes, B8NFF2 (PKS; backbone enzyme encoded by AFLA_062860) and B8NFE8 (encoded by AFLA_062820) in cluster 20, were found to be acetylated at two Kac sites in each enzyme (Lys494 and Lys897 in B8NFF2, and Lys2019 and Lys2423 in B8NFE8). Another backbone enzyme in cluster 21 (NRPS; B8NI19 encoded by AFLA_064240) was acetylated at four sites (Lys819, Lys985, Lys1714, and Lys1800) (S1 Table).

### Proteins involved in pathogenicity and possible roles of Kac in *A*. *flavus*-crop interaction

*A*. *flavus* is a saprophytic filamentous fungal pathogen of oil-rich seeds of various crop species at pre- and post-harvest stages due to its production of aflatoxin [76]. As a serious contaminant of crop production, genes in aflatoxin-specific biosynthetic pathways associated with pathogenicity have been extensively studied [77, 78]. A recent study demonstrated that proteins involved in epigenetic modification, transcription factors, signaling sensing and oxidative-stress response might also affect *A*. *flavus* pathogenicity when colonizing crop seeds. Additionally, the global epigenetic regulators laeA and veA can affect the production of lipase required for host lipid depletion during cell penetration and seed colonization, thereby influencing pathogenicity upon *A*. *flavus* infection of peanut and maize seeds [24]. Recent studies also showed that histone modification, including the histone acetyltransferase AflGcnE [79] and the histone methyltransferase aflrmtA [80], affect the *A*. *flavus* pathogenicity during colonization of maize and peanut seeds. Except for pathway-specific Zn(II)_2_Cys_6_ transcriptional regulator aflR [78], Zhuang et al [81] indicated that the C_2_H_2_ zinc finger transcription factor *mtfA* also governs *A*. *flavus* aflatoxin production and pathogenicity. Among molecules and genes involved in signal sensing in *A*. *flavus*, the most important factors are oxylipins encoded by dioxygenase genes (*ppo* genes). They possibly play a role in pathogenicity, given that loss of oxylipin genes in *A*. *flavus* has been associated with altered pathogenicity on host seeds. Additionally, oxylipins might also potentially be involved in fungus-host cross-communication [82], and there is accumulating evidence that oxylipins are sensed by G protein-coupled receptors [83]. It was also demonstrated that oxidative stress plays the pivotal role in controlling the regulation of morphological transitions and pathogenicity onset in *Aspergillus* sp. Furthermore, the biosynthesis of aflatoxin associated with pathogenecity can be affected by reactive oxygen species regulated by transcription factors such as AtfB, MsnA and SrrA [84].

*A*. *flavus* colonization in crops causes significant economic losses due to destroyed/reduced utilization of aflatoxin-contaminated grains [85]. Aflatoxin contamination, for example in peanuts (*Arachis hypogaea* L.), is the results of a systemic interaction between host plant and *A*. *flavus*. Previous research identified differentially expressed proteins and PTMs during the plant-pathogen interaction process. Additionally, during *A*. *flavus* infection in peanuts, several genes, proteins, and other regulators associated with *A*. *flavus* colonization and peanut resistance to aflatoxin contamination have been identified [76, 86]. Proteomic analyses of interactions between *Fusarium graminearum* and *Triticum aestivum* identify differentially accumulated proteins from both *F*. *graminearum* and *wheat*, with eight proteins from *F*. *graminearum* and functioning mainly in antioxidation (superoxide dismutase and flavin oxidoreductase) and carbon acquisition (fructose-1, 6-bisphosphate aldolase and glyceraldehyde 3-phosphate dehydrogenase) from wheat through glycolysis in a compatible interaction between *F*. *graminearum* and wheat. Previous studies revealed that *F*. *graminearum* directly interacts with wheat in two pathways: antioxidation and glycolysis. During these pathways, the pathogen overcomes oxidative burst and obtains its nutrition supply from wheat [87]. In *F*. *graminearum*, other proteins that might function in fungal-plant interactions include xylanse, protease, cutinase, and cytochrome P450 [88]. Recent studies also revealed that in addition to the quantity of protein synthesis, PTMs are also critical to pathogen invasion. In *Arabidopsis*, pathogen infection is associated with histone deacetylase/ methylation [89], protein phosphorylation also participates in the process of plant-pathogen interaction, and in rice (*Oryza sativa* L.), differentially phosphorylated proteins were identified following *Xanthomonas oryzae pv*. *oryzae* (*Xoo*) infection [90]. Our results revealed several proteins with different Kac sites and involved in nutrient acquisition, including neutral protease (B8NJB2 with two Kac sites), alkaline protease (B8N106 with three Kac sites), enzymes involved in glycolysis (discussed previously), and proteins associated with antioxidation, such as Cu,Zn-superoxide dismutase (B8NUD8 with two Kac sites), glutathione peroxidase (B8MY54 with three Kac sites), and catalase (B8N244 with four Kac sites). These results might suggest that interactions between *A*. *flavus* and crops both in the antioxidant and glycolysis pathways are similar to those between *F*. *graminearum* and wheat. However, the function of Kac in *A*. *flavus*-crop interactions has not been previously reported. As previously discussed, Kac at specific sites in proteins can alter protein nature and ultimately provide modified protein with new functions related to enzyme activity, substrate specificity, structure stability or intracellular localization [90]. Therefore, the Kac might alter the activity of proteins involved in pathogenesis (enzymes in nutritional assimilation, oxidative stress response, aflatoxin-specific pathways, epigenetic modifications, transcription factors, and signaling proteins) and ultimately facilitate colonization and infection process by *A*. *flavus*. Each of these proteins and their respective pathways require further investigation to elucidate the epigenetic mechanisms associated with *A*. *flavus* interactions. To comprehensively understand the function of Kac in *A*. *flavus* interactions with crops, quantitative profiling of Kac during infection at different time points should be performed in future work. Additionally, understanding the role of Kac in the *A*. *flavus*-crop interactions involved in aflatoxin contamination might provide potential targets for the prevention and control of crop contamination by *A*. *flavus*.

## Conclusion

In this study, Kac sites in the *A*. *flavus* proteome were identified using affinity enrichment and LC-MS/MS analysis. We identified 1383 unique Kac sites in 652 acetylated proteins involved in a broad range of cellular functions, including gene expression, secondary metabolite synthesis, and cell growth, indicating that Kac might be vital in regulating *A*. *flavus* physiology. Additionally, this is the first report of enzymes directly involved in aflatoxin biosynthesis being acetylated. The acetylated enzymes involved in central metabolism might affect the activities of metabolic enzymes in the precursor-supplied pathways and regulate metabolic flux during aflatoxin biosynthesis, whereas acetylated enzymes involved in the aflatoxin-biosynthetic pathway might have a direct regulatory role in aflatoxin biosynthesis in *A*. *flavus*.

However, owing to the dynamics of Kac in response to various growthfactor stimulations [91] and the occurrence of low occupancy acetylation as a byproduct of normal cellular metabolism, the function of Kac sites on proteins involved in metabolism required validation. Consequently, quantitative profiling of Kac using SILAC [92], Label-free [93], or iTRAQ labeling [91] needs to be performed in the future. Additionally, methods involving mutation of Kac sites could also contribute to understanding the role of acetylation. For example, Kac sites can be mutated to either glutamine (Q) to mimic acetylated K, or R to prevent acetylation [41]. In *A*. *flavus*, determining the roles of lysine-acetylated enzymes in the production of secondary metabolites, including aflatoxin, still requires further investigation to provide additional insight into the regulatory mechanisms associated with aflatoxin biosynthesis.

*A*. *flavus* has been genetically engineered to generate non-aflatoxin producers used for biocontrol of aflatoxin-producing strains by inactivation of key enzymes in biosynthetic pathways. Understanding acetylation modification of metabolism enzymes involved in aflatoxin production could provide a novel approach for highly efficient engineering of non-aflatoxin producing strains. Additionally, uncovering the function of Kac in *A*. *flavus*-crop interactions involved in aflatoxin contamination might provide potential targets for the prevention and control of crop contamination by saprophytic filamentous fungal pathogen *A*. *flavus*. Our results served as an important resource for functional analysis of Kac in *A*. *flavus* physiology and secondary metabolites biosynthesis.

## Supporting information

S1 Fig **(A)** Overview of experimental procedures used in this study. **(B)** Mass error distribution of the identified peptides. **(C)** Peptide length distribution of the Kac peptides.
(TIF)

S2 Fig Examples of representative MS/MS spectra for acetylated peptides. (**A**) Acetyl-peptide _TNSVEK(ac)INALR_, with an acetylation site at Lys50 of pyruvate kinase (B8MWA0); (**B**) acetyl-peptide _NIQK(ac)GIDFVK_, with an acetylation site at Lys408 of malate dehydrogenase (B8MX84); (**C**) acetyl-peptide _GVLFWHEMDQK(ac)IFK_, with an acetylation site at Lys882 of fatty acid synthase β subunit (B8NL80).
(TIF)

S3 Fig (A) GO, and (B) protein domain enrichment analysis of the Kac proteins. (TIF)

S4 Fig Interaction network of Kac proteins. (TIF)

S5 Fig Interactions of Kac proteins involved in glycolysis/gluconeogenesis, aminoacyl-tRNA biosynthesis, oxidative phosphorylation, proteasome activity, and ribosome activity. (TIF)

S6 Fig **(A)** Proteins involved in aflatoxin biosynthesis. The identified lysine-acetylated enzymes and their identifiers are shown in red. **(B)** The acetylpeptide _IK(ac)EFYYR_, with an acetylation site at Lys1285 of *aflA* (B8NL80). **(C)** The acetylpeptide _GNIGYK(ac)EVPR_, with an acetylation site at Lys504 of *aflB* (B8NL81).
(TIF)

S1 Table The identified acetylated proteins and transcriptional factors (TFs). (XLS)

S2 Table The conserved motifs surrounding the acetylated lysines. (XLS)

S3 Table Functional annotation and enrichment analysis of the acetylated proteins. (XLS)

S4 Table The acetylated proteins involved in protein-protein interaction networks. (XLS)

## References

[1] Martin C, Zhang Y. The diverse functions of histone lysine methylation. Nat Rev Mol Cell Bio, 2005, 6(11): 838–849. 16261189 10.1038/nrm1761

[2] Phillips DMP. The presence of acetyl groups in histones. Biochem J, 1963, 87(2): 258. 13943142 10.1042/bj0870258 PMC1201885

[3] Allfrey VG, Faulkner R, Mirsky AE. Acetylation and methylation of histones and their possible role in the regulation of RNA synthesis. P Natl Acad Sci USA, 1964, 51(5): 786–794. 10.1073/pnas.51.5.786 PMC300163 14172992

[4] Finkemeier I, Laxa M, Miguet L, Howden AJ, Sweetlove LJ. Proteins of diverse function and subcellular location are lysine acetylated in *Arabidopsis*. Plant Physiol, 2011, 155(4): 1779–1790. 10.1104/pp.110.171595 21311031 PMC3091095

[5] Zhang K, Zheng S, Yang JS, Chen Y, Cheng Z. Comprehensive profiling of protein lysine acetylation in *Escherichia coli*. J. Proteome Res. 2013, 12 (2): 844–851. 10.1021/pr300912q 23294111

[6] Xiong Y, Peng X, Cheng Z, Liu W, Wang GL. A comprehensive catalog of the lysine-acetylation targets in rice (*Oryza sativa*) based on proteomic analyses. J Proteomics, 2016, 138: 20–29. 10.1016/j.jprot.2016.01.019 26836501

[7] Colak G, Xie Z, Zhu AY, Dai L, Lu Z, Zhang Y, et al Identification of lysine succinylation substrates and the succinylation regulatory enzyme CobB in *Escherichia coli*. Mol Cell Proteomics, 2013, 12(12): 3509–3520. 10.1074/mcp.M113.031567 24176774 PMC3861704

[8] Weinert BT, Iesmantavicius V, Wagner SA, Schölz C, Gummesson B, Beli P, et al Acetyl-phosphate is a critical determinant of lysine acetylation in *E*. *coli*. Mol Cell, 2013, 51(2): 265–272. 10.1016/j.molcel.2013.06.003 23830618

[9] Kuhn ML, Zemaitaitis B, Hu LI, Sahu A, Sorensen D, Minasov G, et al Structural, kinetic and proteomic characterization of acetyl phosphate-dependent bacterial protein acetylation. PloS One, 2014, 9(4): e94816 10.1371/journal.pone.0094816 24756028 PMC3995681

[10] Kim D, Yu BJ, Kim J, Lee YJ, Choi SG, Kang S, et al The acetylproteome of gram-positive model bacterium *Bacillus subtilis*. Proteomics, 2013, 13(10–11): 1726–1736. 10.1002/pmic.201200001 23468065

[11] Pan J, Ye Z, Cheng Z, Peng X, Wen L, Zhao F. Systematic analysis of the lysine acetylome in *Vibrio parahemolyticus*. J Proteome Res, 2014, 13(7): 3294–3302. 10.1021/pr500133t 24874924

[12] Huang D, Li ZH, You D, Zhou Y, Ye BC. Lysine acetylproteome analysis suggests its roles in primary and secondary metabolism in *Saccharopolyspora erythraea*. Appl Microbiol Biot, 2015, 99(3): 1399–1413. 10.1007/s00253-014-6144-2 25487885

[13] Liao G, Xie L, Li X, Cheng Z, Xie J. Unexpected extensive lysine acetylation in the trump-card antibiotic producer *Streptomyces roseosporus* revealed by proteome-wide profiling. J Proteomics, 2014, 106: 260–269. 10.1016/j.jprot.2014.04.017 24768905

[14] Xie L, Wang X, Zeng J, Zhou M, Duan X, Li Q, et al Proteome-wide lysine acetylation profiling of the human pathogen *Mycobacterium tuberculosis*. Int J Biochem Cell B, 2015, 59: 193–202. 10.1016/j.biocel.2014.11.010 25456444

[15] Henriksen P, Wagner SA, Weinert BT, Sharma S, Bačinskaja G, Rehman M, et al Proteome-wide analysis of lysine acetylation suggests its broad regulatory scope in *Saccharomyces cerevisiae*. Mol Cell Proteomics, 2012, 11(11): 1510–1522. 10.1074/mcp.M112.017251 22865919 PMC3494197

[16] Wu X, Oh MH, Schwarz EM, Larue CT, Sivaguru M, Imai BS, et al Lysine acetylation is a widespread protein modification for diverse proteins in *Arabidopsis*. Plant Physiol, 2011, 155(4): 1769–1778. 10.1104/pp.110.165852 21311030 PMC3091122

[17] Nallamilli BRR, Edelmann MJ, Zhong X, Tan F, Mujahid H, Zhang J, et al Global analysis of lysine acetylation suggests the involvement of protein acetylation in diverse biological processes in rice (*Oryza sativa*). PLoS One, 2014, 9(2): e89283 10.1371/journal.pone.0089283 24586658 PMC3930695

[18] Kim G W, Yang X J. Comprehensive lysine acetylomes emerging from bacteria to humans. Trends Biochem Sci, 2011, 36 (4): 211–220. 10.1016/j.tibs.2010.10.001 21075636

[19] Reyes-Dominguez Y, Narendja F, Berger H, Gallmetzer A, Fernandez-Martin R, Garcia I, et al Nucleosome positioning and histone H3 acetylation are independent processes in the *Aspergillus nidulans* prnD-prnB bidirectional promoter. Eukaryot Cell, 2008, 7(4): 656–663. 10.1128/EC.00184-07 18296621 PMC2292632

[20] Hedtke M, Rauscher S, Röhrig J, Rodríguez-Romero J, Yu Z, Fischer R. Light-dependent gene activation in *Aspergillus nidulans* is strictly dependent on phytochrome and involves the interplay of phytochrome and white collar-regulated histone H3 acetylation. Mol Microbiol, 2015, 97(4): 733–745. 10.1111/mmi.13062 25980340

[21] Nützmann H W, Reyes-Dominguez Y, Scherlach K, Schroeckh V, Horn F, Gacek A, et al Bacteria-induced natural product formation in the fungus *Aspergillus nidulans* requires Saga/Ada-mediated histone acetylation. P Natl Acad Sci USA, 2011, 108(34): 14282–14287. 10.1073/pnas.1103523108 PMC3161617 21825172

[22] Bok JW, Soukup AA, Chadwick E, Chiang YM, Wang CC, Keller NP. VeA and MvlA repression of the cryptic orsellinic acid gene cluster in *Aspergillus nidulans* involves histone 3 acetylation. Mol Microbiol, 2013, 89(5): 963–974. 10.1111/mmi.12326 23841751 PMC3773851

[23] Kale SP, Milde L, Trapp MK, Frisvad JC, Keller NP, Bok JW. Requirement of LaeA for secondary metabolism and sclerotial production in *Aspergillus flavus*. Fungal Genet Biol, 2008, 45(10): 1422–1429. 10.1016/j.fgb.2008.06.009 18667168 PMC2845523

[24] Amaike S, Keller NP. Distinct roles for VeA and LaeA in development and pathogenesis of *Aspergillus flavus*. Eukaryot Cell, 2009, 8(7): 1051–1060. 10.1128/EC.00088-09 19411623 PMC2708460

[25] Chang P K, Scharfenstein L L, Ehrlich K C, Wei Q, Bhatnagar D, Ingber BF. Effects of *laeA* deletion on *Aspergillus flavus* conidial development and hydrophobicity may contribute to loss of aflatoxin production. Fungal biology, 2012, 116(2): 298–307. 10.1016/j.funbio.2011.12.003 22289775

[26] Payne GP, Brown MP. Genetics and physiology of aflatoxin biosynthesis. Annu Rev Phytopathol. 1998, 36, 329–362. 10.1146/annurev.phyto.36.1.329 15012504

[27] Chang PK, Scharfenstein LL, Mack B, Yu J, Ehrlich KC. Transcriptomic profiles of *Aspergillus flavus* CA42, a strain that produces small sclerotia, by decanal treatment and after recovery. Fungal Genet Biol, 2014, 68: 39–47. 10.1016/j.fgb.2014.04.007 24780887

[28] Bayman P, Cotty PJ. Genetic diversity in *Aspergillus flavus*: association with aflatoxin production and morphology. Can J Bot, 1993, 71(1): 23–31.

[29] Wu X, Zhou B, Yin C, Guo Y, Lin Y, Pan L, et al Characterization of natural antisense transcript, sclerotia development and secondary metabolism by strand-specific RNA sequencing of *Aspergillus flavus*. PloS one, 2014, 9(5): e97814 10.1371/journal.pone.0097814 24849659 PMC4029826

[30] Vizcaíno JA, Csordas A, del-Toro N, Dianes JA, Griss J, Lavidas I, et al 2016 update of the PRIDE database and related tools. Nucleic Acids Res, 2016, 44 (D1): D447–D456. 10.1093/nar/gkv1145 26527722 PMC4702828

[31] Cox J, Mann M. MaxQuant enables high peptide identification rates, individualized ppb-range mass accuracies and proteome-wide protein quantification. Nat Biotechnol, 2008, 26 (12): 1367–1372. 10.1038/nbt.1511 19029910

[32] Wu J, Mao X, Cai T, Luo J, Wei L. KOBAS server: a web-based platform for automated annotation and pathway identification. Nucleic Acids Res, 2006, 34: W720–W724. 10.1093/nar/gkl167 16845106 PMC1538915

[33] Zdobnov EM, Apweiler R. InterProScan an integration platform for the signature-recognition methods in InterPro. Bioinformatics, 2001, 17: 847–848. 11590104 10.1093/bioinformatics/17.9.847

[34] Chou MF, Schwartz D. Biological sequence motif discovery using motif-x, Curr Protoc Bioinformatics 13 (2011) 15–24. 10.1002/0471250953.bi1315s35 21901740

[35] Shannon P, Markiel A, Ozier O, Baliga NS, Wang JT, Ramage D, et al Cytoscape: a software environment for integrated models of biomolecular interaction networks. Genome Res, 2003, 13 (11): 2498–2504. 10.1101/gr.1239303 14597658 PMC403769

[36] Zhang J, Sprung R, Pei J, Tan X, Kim S, Zhu H, et al Lysine acetylation is a highly abundant and evolutionarily conserved modification in *Escherichia coli*. Mol Cell Proteomics, 2009, 8(2): 215–225. 10.1074/mcp.M800187-MCP200 18723842 PMC2634580

[37] Kim SC, Sprung R, Chen Y, Xu Y, Ball H, Pei J, et al Substrate and functional diversity of lysine acetylation revealed by a proteomics survey. Mol Cell, 2006, 23(4): 607–618. 10.1016/j.molcel.2006.06.026 16916647

[38] Li D, Lv B, Tan L, Yang Q, Liang W. Acetylome analysis reveals the involvement of lysine acetylation in diverse biological processes in *Phytophthora sojae*. Sci Rep, 2016, 6: 29897 10.1038/srep29897 27412925 PMC4944153

[39] Lv B, Yang Q, Li D, Liang W, Song L. Proteome-wide analysis of lysine acetylation in the plant pathogen *Botrytis cinerea*. Sci Rep, 2016, 6: 29313 10.1038/srep29313 27381557 PMC4933888

[40] Liu L, Wang G, Song L, Lv B, Liang W. Acetylome analysis reveals the involvement of lysine acetylation in biosynthesis of antibiotics in *Bacillus amyloliquefaciens*. Sci Rep, 2016, 6: 20108 10.1038/srep20108 26822828 PMC4731788

[41] Choudhary C, Kumar C, Gnad F, Nielsen ML, Rehman M, Walther TC, et al Lysine acetylation targets protein complexes and co-regulates major cellular functions. Science 2009, 325, 834–840. 10.1126/science.1175371 19608861

[42] Yang XJ. Lysine acetylation and the bromodomain: a new partnership for signaling. Bioessays, 2004, 26(10): 1076–1087. 10.1002/bies.20104 15382140

[43] Zhao S, Xu W, Jiang W, Yu W, Lin Y, Zhang T, et al Regulation of cellular metabolism by protein lysine acetylation. Science, 2010, 327(5968): 1000–1004. 10.1126/science.1179689 20167786 PMC3232675

[44] Strauss J, Reyes-Dominguez Y. Regulation of secondary metabolism by chromatin structure and epigenetic codes. Fungal Genet Biol, 2011, 48(1): 62–69. 10.1016/j.fgb.2010.07.009 20659575 PMC3935439

[45] Brosch G, Loidl P, Graessle S. Histone modifications and chromatin dynamics: a focus on filamentous fungi. FEMS Microbiol Rev, 2008, 32(3): 409–439. 10.1111/j.1574-6976.2007.00100.x 18221488 PMC2442719

[46] Park JM, Jo SH, Kim MY, Kim TH, Ahn YH. Role of transcription factor acetylation in the regulation of metabolic homeostasis. Protein Cell, 2015, 6(11): 804–813. 10.1007/s13238-015-0204-y 26334401 PMC4624674

[47] Schröder S, Herker E, Itzen F, He D, Thomas S, Gilchrist DA, et al Acetylation of RNA polymerase II regulates growth-factor-induced gene transcription in mammalian cells. Mol Cell, 2013, 52(3): 314–324. 10.1016/j.molcel.2013.10.009 24207025 PMC3936344

[48] Thompson PR, Wang D, Wang L, Fulco M, Pediconi N, Zhang D, et al Regulation of the p300 HAT domain via a novel activation loop. Nat Struct Mol Biol, 2004, 11(4): 308–315. 10.1038/nsmb740 15004546

[49] Grant PA, Duggan L, Côté J, Roberts SM, Brownell JE, Candau R, et al Yeast Gcn5 functions in two multisubunit complexes to acetylate nucleosomal histones: characterization of an Ada complex and the SAGA (Spt/Ada) complex. Gene Dev, 1997, 11(13): 1640–1650. 9224714 10.1101/gad.11.13.1640

[50] Delpech M, Levy-Favatier F, Kruh J. In vitro, non enzymatic labelling of histone H1 with [^14^C] acetyl CoA. Biochimie, 1983, 65:291–294. 6409164 10.1016/s0300-9084(83)80281-8

[51] Weinert BT, Moustafa T, Iesmantavicius V, Zechner R, Choudhary C. Analysis of acetylation stoichiometry suggests that SIRT3 repairs nonenzymatic acetylation lesions. EMBO J, 2015, 34(21): 2620–2632. 10.15252/embj.201591271 26358839 PMC4641529

[52] Gao X, Hong H, Li WC, Yang L, Huang J, Xiao YL, et al Downregulation of rubisco activity by non-enzymatic acetylation of RbcL. Mol Plant, 2016, 9(7): 1018–1027. 10.1016/j.molp.2016.03.012 27109602

[53] Wolfe AJ. The acetate switch. Microbiol Mol Biol R, 2005, 69(1): 12–50. 10.1128/MMBR.69.1.12-50.2005 PMC1082793 15755952

[54] Morrison DK. The 14-3-3 proteins: integrators of diverse signaling cues that impact cell fate and cancer development. Trends Cell Biol, 2009, 19(1): 16–23. 10.1016/j.tcb.2008.10.003 19027299 PMC3073487

[55] Yang XJ, Seto E. Lysine acetylation: codified crosstalk with other posttranslational modifications. Molecular cell, 2008, 31(4): 449–461. 10.1016/j.molcel.2008.07.002 18722172 PMC2551738

[56] Pajares MA, Markham GD. Methionine adenosyltransferase (S-adenosylmethionine synthetase). Advances in Enzymology and Related Areas of Molecular Biology, 2011, 78: 449 22220481 10.1002/9781118105771.ch11

[57] Markham GD, Pajares MA. Structure-function relationships in methionine adenosyltransferases. Cell Mol Life Sci, 2009, 66(4): 636–648. 10.1007/s00018-008-8516-1 18953685 PMC2643306

[58] Wijayasinghe YS, Blumenthal RM, Viola RE. Producing proficient methyl donors from alternative substrates of S-adenosylmethionine synthetase. Biochemistry, 2014, 53(9): 1521–1526. 10.1021/bi401556p 24528526 PMC3985469

[59] Lu SC, Mato JM. S-adenosylmethionine in liver health, injury, and cancer. Physiol Rev, 2012, 92: 1515–1542. 10.1152/physrev.00047.2011 23073625 PMC3698976

[60] Sun M, Guo H, Lu G, Gu J, Wang X, Zhang XE, et al Lysine acetylation regulates the activity of *Escherichia coli* S-adenosylmethionine synthase. Acta Bioch Bioph Sin, 2016, 48(8): 723–731. 10.1093/abbs/gmw066 27421658

[61] Sadoul K, Khochbin S. The growing landscape of tubulin acetylation: lysine 40 and many more. Biochem J, 2016, 473(13): 1859–1868. 10.1042/BCJ20160172 27354562

[62] Andrianopoulos A, Kourambas S, Sharp JA, Davis MA, Hynes MJ. Characterization of the *Aspergillus nidulans nmrA* gene involved in nitrogen metabolite repression. J Bacteriol, 1998, 180(7): 1973–1977. 9537404 10.1128/jb.180.7.1973-1977.1998 PMC107119

[63] Si H, Rittenour W, Harris S. Roles of *Aspergillus nidulans* Cdc42/Rho GTPase regulators in hyphal morphogenesis and development. Mycologia, 2016: 15–232. 10.3852/15-232 26932184

[64] Seo JA, Han KH, Yu JH. Multiple roles of a heterotrimeric G-protein γ-subunit in governing growth and development of *Aspergillus nidulans*. Genetics, 2005, 171(1): 81–89. 10.1534/genetics.105.042796 15944346 PMC1456535

[65] Noventa-Jordão MA, do Nascimento AM, Goldman MHS, Terenzi HF, Goldman GH. Molecular characterization of ubiquitin genes from *Aspergillus nidulans*: mRNA expression on different stress and growth conditions. BBA-Gene Struct Exp, 2000, 1490(3): 237–244. 10.1016/s0167-4781(99)00242-0 10684969

[66] Wang Q, Zhang Y, Yang C, Xiong H, Lin Y, Yao J, et al Acetylation of metabolic enzymes coordinates carbon source utilization and metabolic flux. Science, 2010, 327(5968): 1004–1007. 10.1126/science.1179687 20167787 PMC4183141

[67] Starai VJ, Escalante-Semerena J.C. Identification of the protein acetyltransferase (Pat) enzyme that acetylates acetyl-CoA synthetase in *Salmonella enterica*. J Mol Biol, 2004, 340(5): 1005–1012. 10.1016/j.jmb.2004.05.010 15236963

[68] Minto RE, Townsend CA. Enzymology and molecular biology of aflatoxin biosynthesis. Chem Rev, 1997, 97(7): 2537–2556. 11851470 10.1021/cr960032y

[69] Xing S, Poirier Y. The protein acetylome and the regulation of metabolism. Trends Plant Sci, 2012, 17(7): 423–430. 10.1016/j.tplants.2012.03.008 22503580

[70] Ehrlich KC, Li P, Scharfenstein L, Chang PK. HypC, the anthrone oxidase involved in aflatoxin biosynthesis. Appl Environ Microb, 2010, 76: 3374–3377. 10.1128/AEM.02495-09 PMC2869142 20348292

[71] Brown DW, Adams TH, Keller NP. *Aspergillus* has distinct fatty acid synthasis for primary and secondary metabolism. The National Academy of Sciences of the USA, 1996, 93(25): 14873–1477. 10.1073/pnas.93.25.14873 PMC26229 8962148

[72] Starai VJ, Celic I, Cole RN, Boeke JD, Escalante-Semerena JC. Sir2-dependent activation of acetyl-CoA synthetase by deacetylation of active lysine. Science, 2002, 298(5602): 2390–2392. 10.1126/science.1077650 12493915

[73] Khaldi N, Seifuddin FT, Turner G, Haft D, Nierman WC, Wolfe KH, et al SMURF: genomic mapping of fungal secondary metabolite clusters. Fungal Genet. Biol. 2010, 47:736–41. 10.1016/j.fgb.2010.06.003 20554054 PMC2916752

[74] Georgianna D, Fedorova ND, Burroughs JL, Dolezal AL, Bok JW, Horowitz-brown S, et al Beyond aflatoxin: four distinct expression patterns and functional roles associated with *Aspergillus flavus* secondary metabolism gene clusters. Mol Plant Pathol, 2010, 11(2): 213–226. 10.1111/j.1364-3703.2009.00594.x 20447271 PMC4116135

[75] Amaike S, Keller NP. Aspergillus flavus. Annu Rev Phytopathol, 2011, 49: 107–133. 10.1146/annurev-phyto-072910-095221 21513456

[76] Wang H, Lei Y, Yan L, Wan L, Ren X, Chen S, et al Functional genomic analysis of *Aspergillus flavus* interacting with resistant and susceptible peanut. Toxins, 2016, 8(2). 10.3390/toxins8020046 PMC4773799 26891328

[77] Yu J, Chang PK, Ehrlich KC, Cary JW, Bhatnagar D, Cleveland TE, et al Clustered pathway genes in aflatoxin biosynthesis. Applied Environ Microb, 2004, 70(3): 1253–1262. 10.1128/AEM.70.3.1253-1262.2004 PMC368384 15006741

[78] Chang PK. Lack of interaction between AFLR and AFLJ contributes to nonaflatoxigenicity of *Aspergillus sojae*. Journal Biotechnol, 2004, 107(3): 245–253. 10.1016/j.jbiotec.2003.10.012 14736460

[79] Lan H, Sun R, Fan K, Yang K, Zhang F, Nie XY, et al The *Aspergillus flavus* histone acetyltransferase *AflGcnE* regulates morphogenesis, aflatoxin biosynthesis, and pathogenicity. Frontiers Microbiol, 2016, 7:1324. 10.3389/fmicb.2016.01324 PMC5003836 27625637

[80] Li Y, He Y, Li X, Fasoyin OE, Hu Y, Liu Y, et al Histone Methyltransferase *aflrmtA* gene is involved in the morphogenesis, mycotoxin biosynthesis, and pathogenicity of *Aspergillus flavus*. Toxicon, 2017, 127: 112–121. 10.1016/j.toxicon.2017.01.013 28109854

[81] Zhuang Z, Lohmar JM, Satterlee T, Cary JW, Calvo AM. The master transcription factor *mtfA* governs aflatoxin production, morphological development and pathogenicity in the fungus *Aspergillus flavus*. Toxins, 2016, 8(1): 29. 10.3390/toxins8010029 PMC4728551 26805883

[82] Brown SH, Scott JB, Bhaheetharan J, Sharpee WC, Milde L, Wilson RA, et al Oxygenase coordination is required for morphological transition and the host-fungus interaction of *Aspergillus flavus*. Mol Plant-Microbe In, 2009, 22(7): 882–894. 10.1094/MPMI-22-7-0882 19522570

[83] Affeldt KJ, Brodhagen M, Keller NP. *Aspergillus* oxylipin signaling and quorum sensing pathways depend on G protein-coupled receptors. Toxins, 2012, 4(9): 695–717. 10.3390/toxins4090695 23105976 PMC3475224

[84] Amare MG, Keller NP. Molecular mechanisms of *Aspergillus flavus* secondary metabolism and development. Fungal Genet Biol, 2014, 66: 11–18. 10.1016/j.fgb.2014.02.008 24613992

[85] Wang H, Lei Y, Yan L, Cheng K, Dai X, Wang L, et al Deep sequencing analysis of transcriptomes in *Aspergillus flavus* in response to resveratrol. BMC Microbiol, 2015, 15(1):182. 26420172 10.1186/s12866-015-0513-6 PMC4589122

[86] Wang Z, Yan S, Liu C, Chen F, Wang T. Proteomic analysis reveals an aflatoxin-triggered immune response in cotyledons of *Arachis hypogaea* infected with *Aspergillus flavus*. J Proteome Res, 2012, 11(5): 2739–2753. 10.1021/pr201105d 22424419

[87] Zhou W, Eudes F, Laroche A. Identification of differentially regulated proteins in response to a compatible interaction between the pathogen *Fusarium graminearum* and its host, *Triticum aestivum*. Proteomics, 2006, 6(16): 4599–4609. 10.1002/pmic.200600052 16858732

[88] Cuomo CA, Güldener U, Xu JR, Trail F, Turgeon BG, Di Pietro A, et al The *Fusarium graminearum* genome reveals a link between localized polymorphism and pathogen specialization. Science, 2007, 317(5843): 1400–1402. 10.1126/science.1143708 17823352

[89] Delapena C, Rangelcano A, Alvarezvenegas R. Regulation of disease-responsive genes mediated by epigenetic factors: interaction of Arabidopsis-Pseudomonas. Mol Plant Pathol, 2012, 13(4): 388–398. 10.1111/j.1364-3703.2011.00757.x 22023111 PMC6638851

[90] Hou Y, Qiu J, Tong X, Wei X, Nallamilli BR, Wu W, et al A comprehensive quantitative phosphoproteome analysis of rice in response to bacterial blight. BMC Plant Biol, 2015, 15(1): 163. 26112675 10.1186/s12870-015-0541-2 PMC4482044

[91] Bryson BD, White FM. Quantitative profiling of lysine acetylation reveals dynamic crosstalk between receptor tyrosine kinases and lysine acetylation. PloS one, 2015, 10(5): e0126242 10.1371/journal.pone.0126242 25978619 PMC4433260

[92] Collier TS, Hawkridge AM, Georgianna DR, Payne GA, Muddiman DC. Top-down identification and quantification of stable isotope labeled proteins from *Aspergillus flavus* using online nano-flow reversed-phase liquid chromatography coupled to a LTQ-FTICR mass spectrometer. Anal Chem, 2008, 80(13): 4994–5001. 10.1021/ac800254z 18512951 PMC3779437

[93] Georgianna DR, Hawkridge AM, Muddiman DC, Payne GA. Temperature-dependent regulation of proteins in *Aspergillus flavus*: whole organism stable isotope labeling by amino acids. J Proteome Res, 2008, 7(7): 2973–2979. 10.1021/pr8001047 18529071

