# Supplementary figures and images for: Proteome-wide profiling of protein lysine acetylation in Aspergillus flavus
Source: PLoS One. 2017 Jun 5;12(6):e0178603. doi: 10.1371/journal.pone.0178603 (PMC5459447; doi:10.1371/journal.pone.0178603)

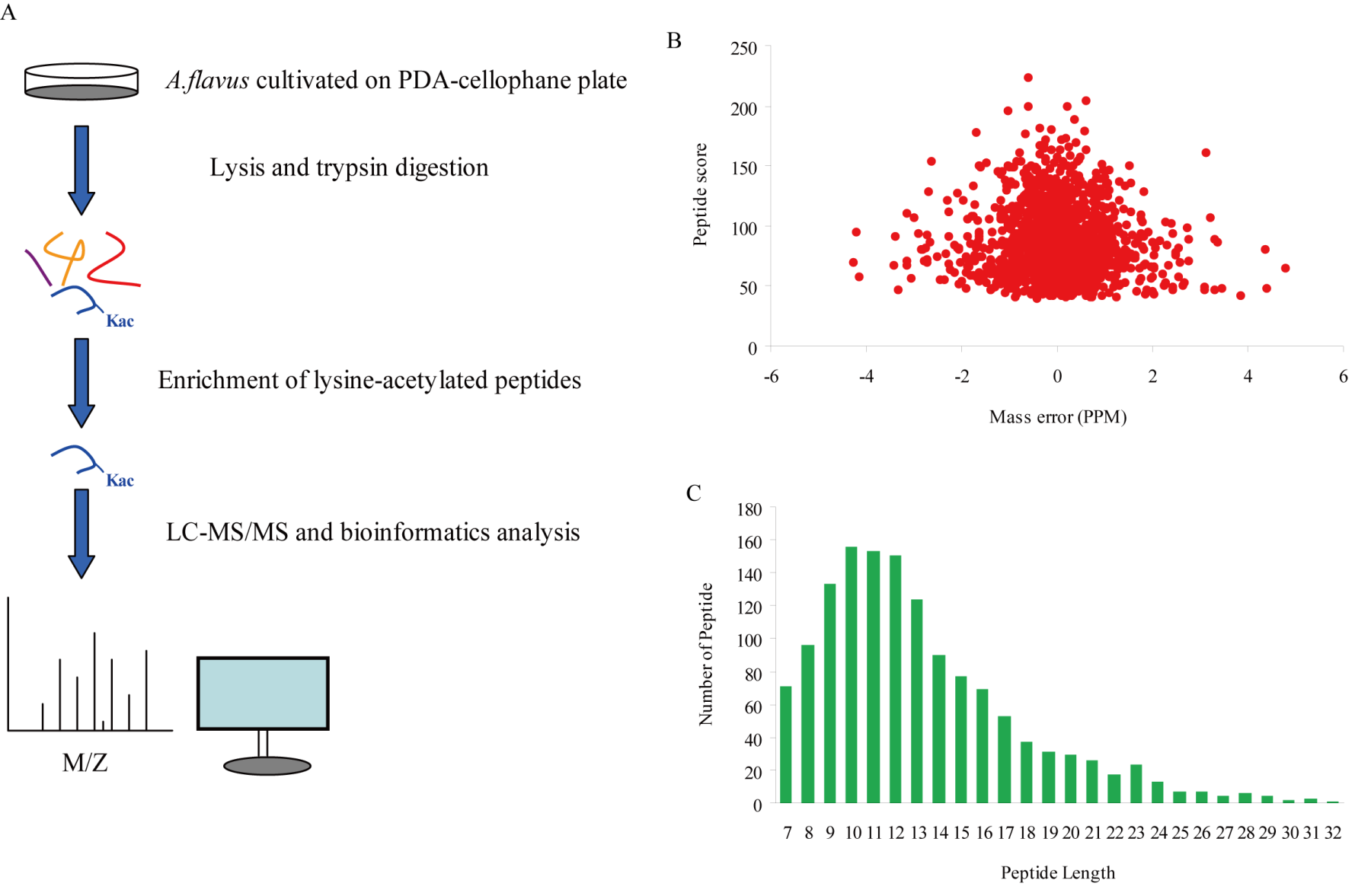

Supplement: S1 Fig — (A) Overview of experimental procedures used in this study. (B) Mass error distribution of the identified peptides. (C) Peptide length distribution of the Kac peptides. (TIF) [file pone.0178603.s001.tif]

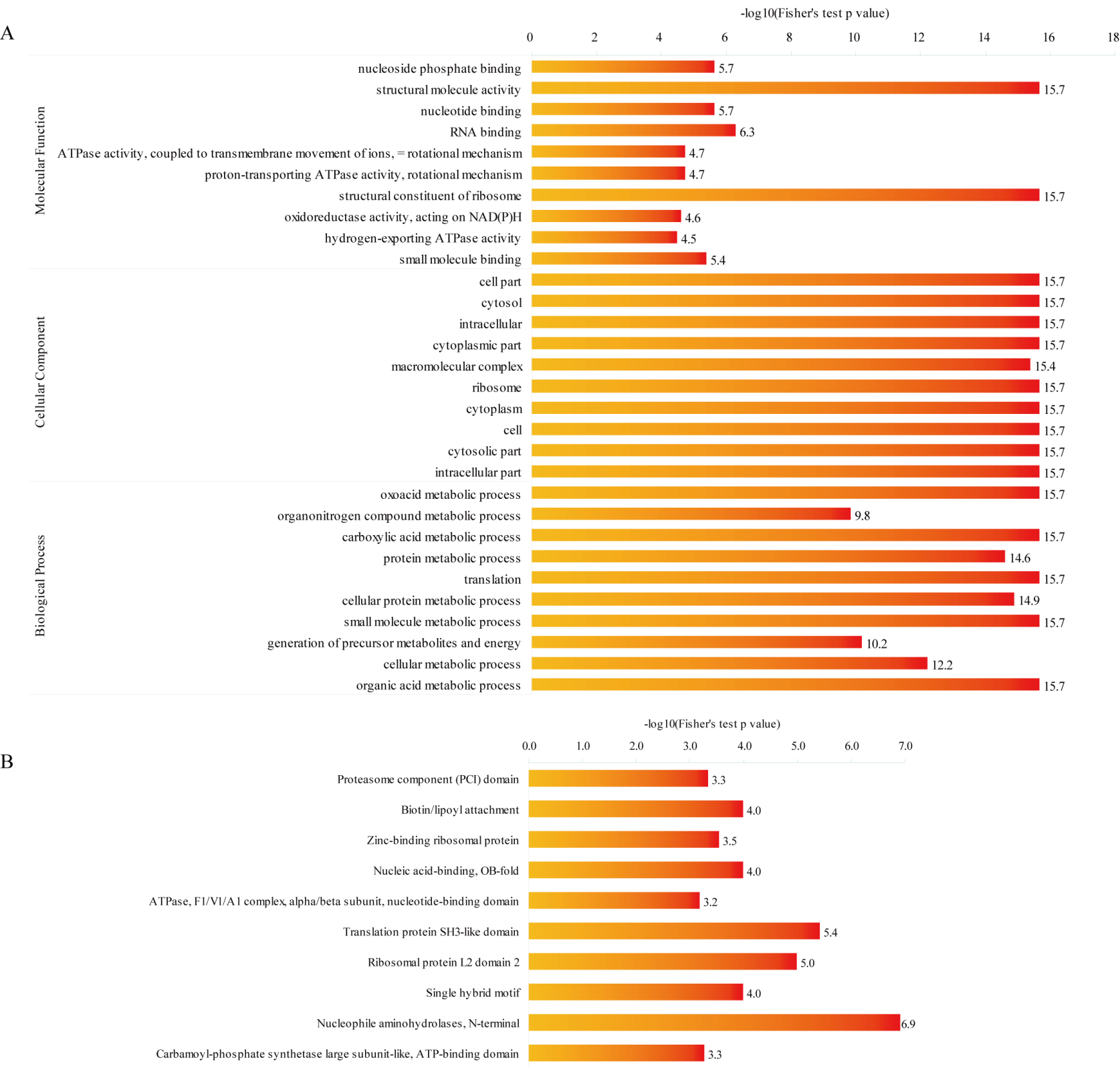

Supplement: S3 Fig — (TIF) [file pone.0178603.s003.tif]

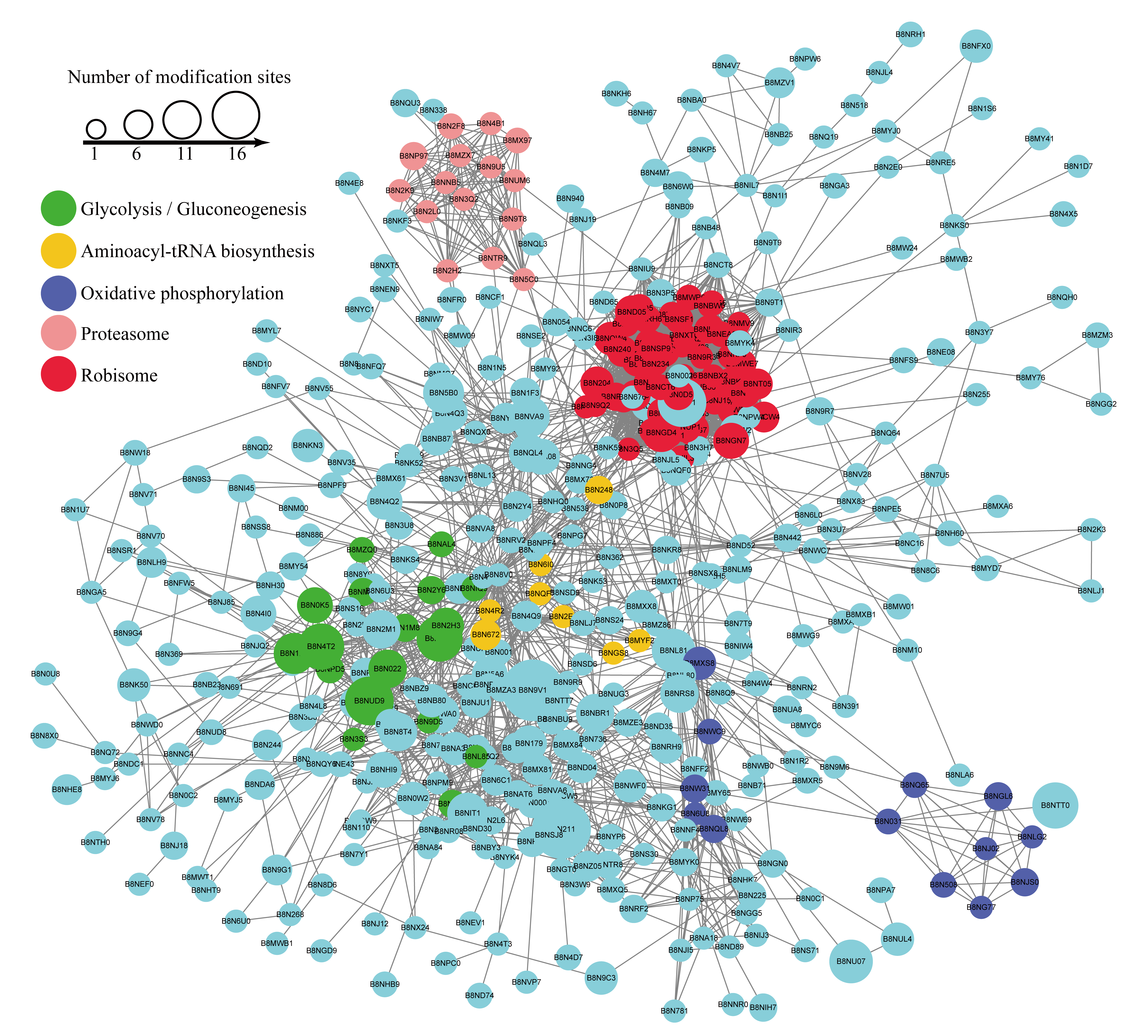

Supplement: S4 Fig — (TIF) [file pone.0178603.s004.tif]

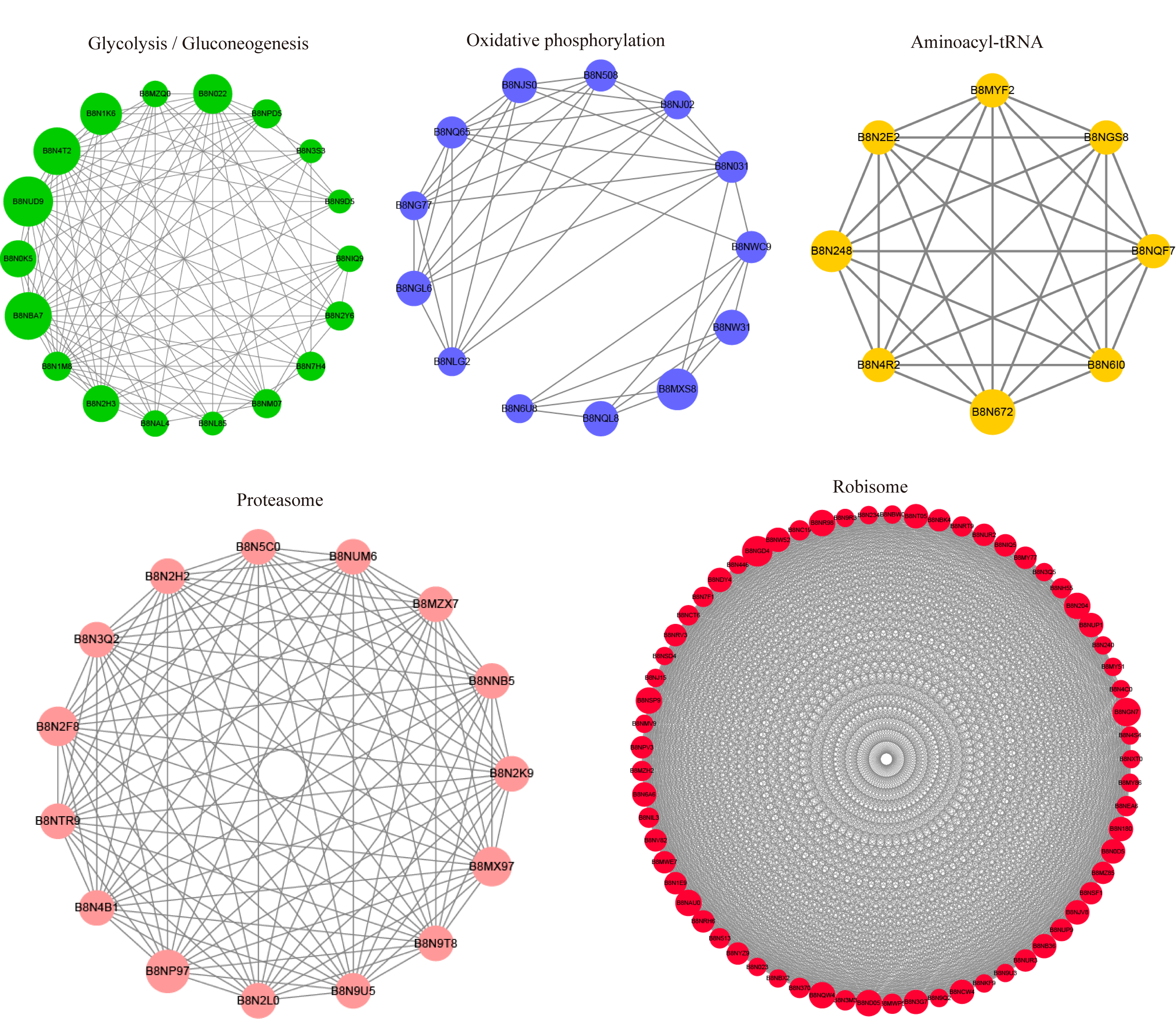

Supplement: S5 Fig — (TIF) [file pone.0178603.s005.tif]

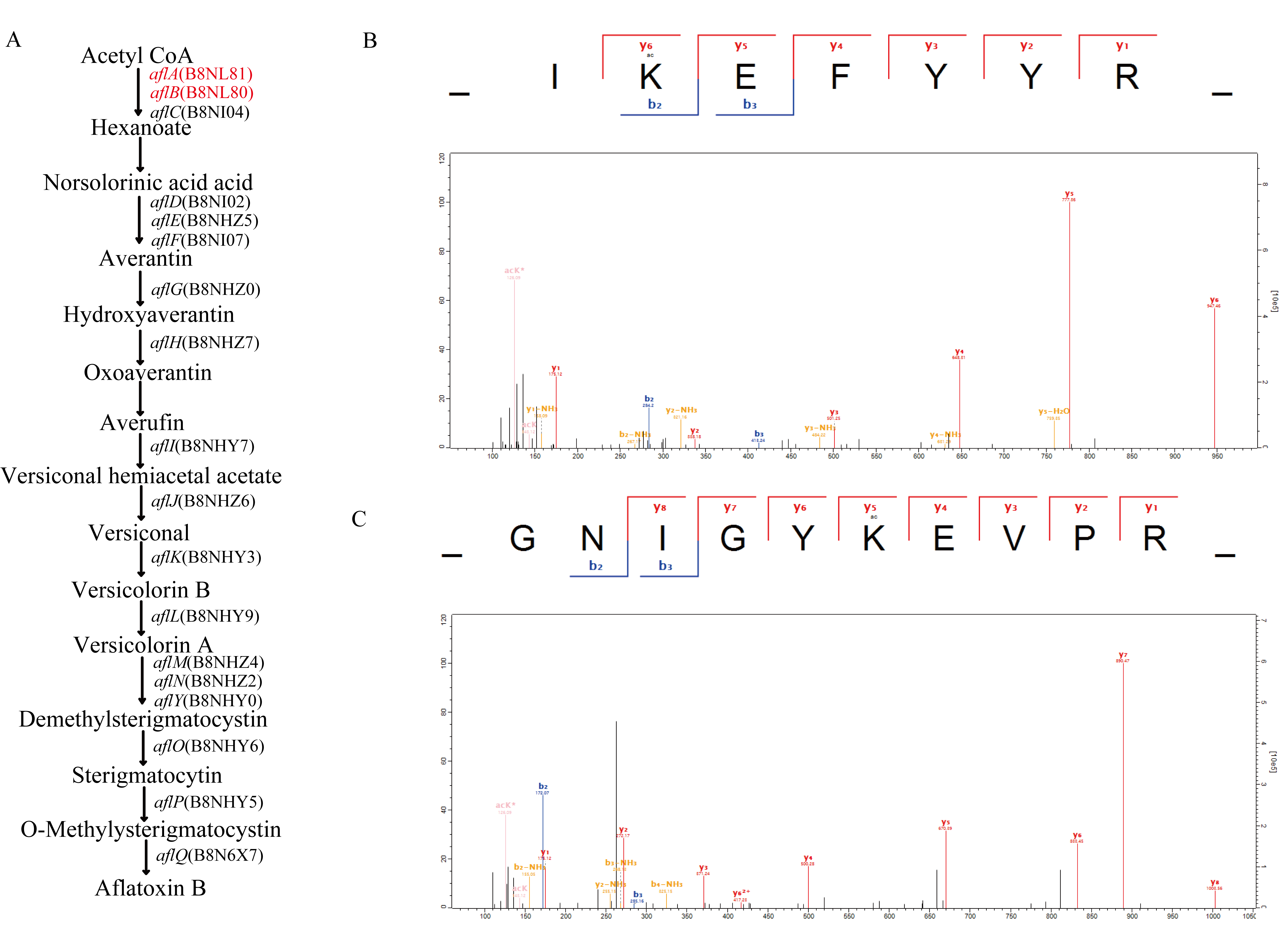

Supplement: S6 Fig — (A) Proteins involved in aflatoxin biosynthesis. The identified lysine-acetylated enzymes and their identifiers are shown in red. (B) The acetylpeptide _IK(ac)EFYYR_, with an acetylation site at Lys1285 of aflA (B8NL80). (C) The acetylpeptide _GNIGYK(ac)EVPR_, with an acetylation site at Lys504 of aflB (B8NL81). (TIF) [file pone.0178603.s006.tif]
